# Quantifying Charge Carrier Localization in PBTTT Using Thermoelectric and Spectroscopic Techniques

**DOI:** 10.1021/acs.jpcc.3c01152

**Published:** 2023-06-14

**Authors:** Shawn A. Gregory, Amalie Atassi, James F. Ponder, Guillaume Freychet, Gregory M. Su, John R. Reynolds, Mark D. Losego, Shannon K. Yee

**Affiliations:** † School of Materials Science and Engineering, Georgia Institute of Technology, Atlanta, Georgia 30332, United States; ‡ George W. Woodruff School of Mechanical Engineering, Georgia Institute of Technology, Atlanta, Georgia 30332, United States; § NSLS-II, Brookhaven National Laboratory, Upton, New York 11973, United States; ∥ Advanced Light Source, Lawrence Berkeley National Laboratory, Berkeley, California 94720, United States; ⊥ Materials Sciences Division, Lawrence Berkeley National Laboratory, Berkeley, California 94720, United States; # School of Chemistry and Biochemistry, Georgia Institute of Technology, Atlanta, Georgia 30332, United States

## Abstract

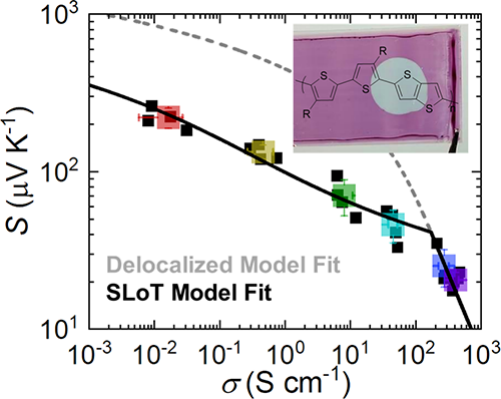

Chemically doped poly[2,5-bis(3-alkylthiophen-2-yl)thieno[3,2-*b*]thiophene] (PBTTT) shows promise for many organic electronic applications, but rationalizing its charge transport properties is challenging because conjugated polymers are inhomogeneous, with convoluted optical and solid-state transport properties. Herein, we use the semilocalized transport (SLoT) model to quantify how the charge transport properties of PBTTT change as a function of iron(III) chloride (FeCl_3_) doping level. We use the SLoT model to calculate fundamental transport parameters, including the carrier density needed for metal-like electrical conductivities and the position of the Fermi energy level with respect to the transport edge. We then contextualize these parameters with other polymer-dopant systems and previous PBTTT reports. Additionally, we use grazing incidence wide-angle X-ray scattering and spectroscopic ellipsometry techniques to better characterize inhomogeneity in PBTTT. Our analyses indicate that PBTTT obtains high electrical conductivities due to its quickly rising reduced Fermi energy level, and this rise is afforded by its locally high carrier densities in highly ordered microdomains. Ultimately, this report sets a benchmark for comparing transport properties across polymer-dopant-processing systems.

## Introduction

**Figure 1:**
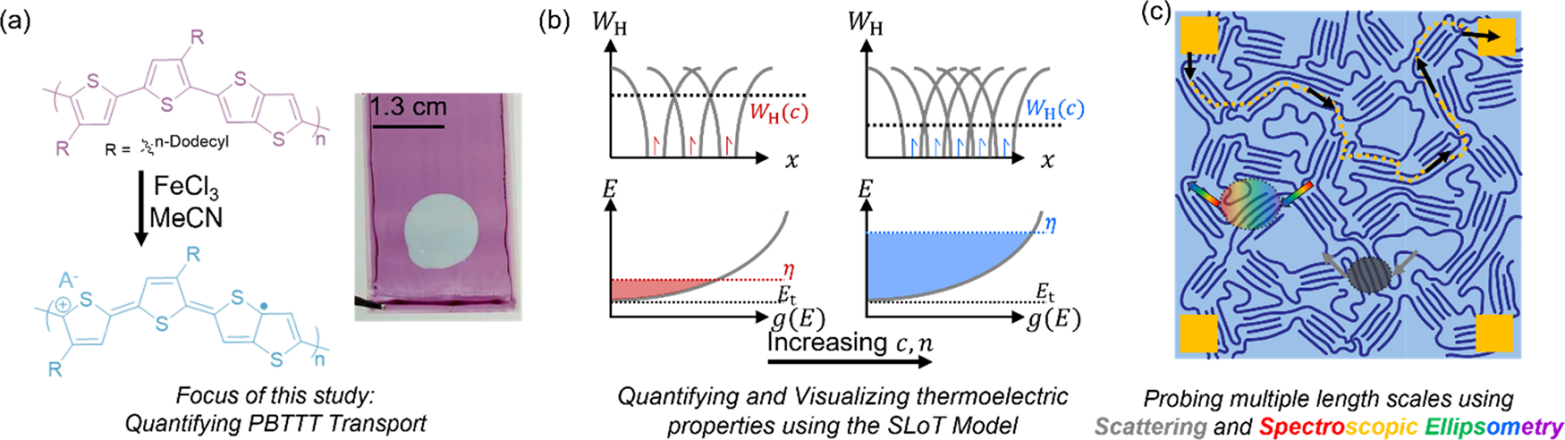
Visual summarizing the key facets of this study. (a) Neutral PBTTT-C12 (purple) is sequentially doped with FeCl_3_ at various concentrations to control the extent of oxidation and resulting thermoelectric properties. Oxidatively doped PBTTT (blue) contains polaronic charge carriers (likely a combination of polarons and bipolarons) and distinctly different electronic and optical properties (see the inset for a digital photograph of neutral PBTTT with a doped circular region). (b) SLoT model is used to contextualize the thermoelectric properties of PBTTT at various doping levels. At low FeCl_3_ concentrations and low extents of oxidation, PBTTT is lightly doped, the density of states, *g*(*E*), is lightly filled with mobile charge carriers and has a low reduced Fermi energy level, η, and the charge carriers are spatially localized with high hopping activation energies, *W*_H_ (see leftmost figures in red). In contrast, at high FeCl_3_ concentrations and high extents of oxidation, PBTTT is heavily doped, the density of states is heavily filled with mobile charge carriers and has a high reduced Fermi energy level, and the charge carriers can be thought as spatially delocalized with little-to-no hopping activation energies. (c) Cartoon illustrating different measurement techniques and their interaction area and conditions. Thermoelectric measurements, represented by gold square contact pads and dashed gold percolated transport pathway, are indicative of the appropriately weighted bulk ensemble average charge transport in all inhomogeneous microdomains along a closed circuit and percolated pathway. In contrast, scattering and spectroscopic measurements can glean insight into microstructure and transport properties in specific microscopic domains that do not require a closed circuit and percolated pathway but may require other physical conditions to be met (e.g., periodic ordering for Bragg diffraction).

Semiconducting polymers are used in a variety of optical and electronic applications because of their synthetic tunability, mechanical compliancy, and processability.^1^ One example is poly[2,5-bis(3-alkylthiophen-2-yl)thieno[3,2-*b*]thiophene] (PBTTT), which shows promise in many applications, such as thermoelectrics^2−4^ and transistors.^5,6^ PBTTT is used in these applications because it is a solution processible conjugated polymer that has remarkably high carrier mobilities (μ ranging from ∼0.1 to 10 cm^2^ V^–1^ s^–1^, measured using multiple techniques)^7−10^ and electrical conductivities (σ, ranging from 10^1^ to ≫10^3^ S cm^–1^),^10−19^ depending on the doping and processing conditions (Figure 1a). Despite these high performing transport properties, it is not fully and quantifiably understood how and to what extent side-chain chemistry, dopants, and other processing conditions affect transport in PBTTT. Quantifying key parameters and characteristics that lead to these macroscopic transport properties is needed for the rational development of polymer-dopant-processing systems.

Charge transport models contextualize macroscopically measurable transport properties to microscopic spatial and energetic distributions and can provide insights into rational design. We have previously demonstrated that a semilocalized transport (SLoT) model can isolate and quantify localized (hopping-like) and delocalized (metal-like) contributions to the measurable electrical conductivity (σ) and Seebeck coefficient (*S*).^20^ Central to charge transport models is the assertion of a transport function; for the SLoT model, the transport function, σ_E_(*E*, *T*, *c*), is given as
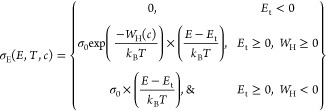
1

This transport function quantifies the contributions of charge carriers at a specific electron energy level (*E*), temperature (*T*), and carrier ratio (*c*, the number of charge carriers per site, which is linearly related to the carrier density, *n*)^20,21^ to the measurable transport properties, σ and *S*.^22−24^Eq 1 states that charge carriers at some energy level (*E*) less than the transport edge (*E*_t_, which can be synonymous with a band edge and the reference zero energy level, see illustrations in Figure 1b) do not contribute to the transport function nor to charge transport. In contrast, charge carriers that have some *E* greater than *E*_t_ contribute meaningfully to charge transport and their contribution is weighted by  σ_0_ is independent of the doping level, can ideally be related to the effective mass and energy independent mobility, and laterally shifts the *S*(σ) curve.^22^ The  term captures the hopping-like contribution to charge transport due to the spatial and electrostatic localization of charge carriers in an inhomogeneous medium, such as a semiconducting polymer like PBTTT (see illustrations in Figure 1b).^25−28^ For example, charge transport is likely greater along the conjugated backbones and between more ordered π – π stacks, rather than between alkyl side chains and less ordered regions.^29−32^*W*_H_(*c*) is the localization energy, and it is calculable from macroscopic transport measurements; therefore, *W*_H_(*c*) represents a bulk ensemble average. *W*_H_(*c*) generally decreases as the carrier ratio, or carrier density, increases because the carriers’ electrostatic potential wells increasingly impinge on one another and the barrier for hopping decreases (see illustrations in Figure 1b).^27,29,33,34^ Eventually, at some carrier ratio, *W*_H_ may be near negligible (≲*k*_B_*T* or zero), but all polymer-dopant-processing systems do not necessarily obtain these low *W*_H_ values.^34−36^ If *W*_H_ ≲ 0, then localization is not the dominant physical contribution that explains σ(*T*), the SLoT model asserts , and the transport function is dependent on only σ_0_ and . Finally,  captures the electron energy-dependent contribution to the transport function, akin to delocalized and metal-like transport formalisms.^22−24^ As the charge carrier ratio and density increase, the energy levels which charge carriers occupy (*E*) and the Fermi energy level (*E*_F_) increase with respect to the transport edge (see illustrations in Figure 1b).

Evaluating the transport function with the Boltzmann transport equation yields expressions that relate the macroscopic and measurable σ and *S* values to these microscopic parameters and distributions detailed in eq 1.^22−24^ The electrical conductivity expression is
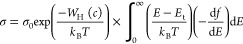
2and eq 2 is a function of the energetic distribution of the charge carriers and their localization. As the extent of doping increases, localization decreases and the energy levels that charge carriers occupy increase, leading to large increases in σ. The Seebeck coefficient expression is
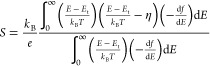
3where , is known as the reduced Fermi energy level, and represents the energetic distance between the Fermi energy level (*E*_F_) and the transport edge (see illustrations in Figure 1b). Notably, eq 3 is only a function of the energetic distribution of the charge carriers and is not a function of σ_0_ nor *W*_H_. Therefore, there are only one η value and one set of Fermi integral values for eq 3 to be true at a fixed and measured Seebeck coefficient.^24^ Furthermore, by measuring σ(*T*) and *S*(*T*) and by calculating the Fermi integral values, *W*_H_ and σ_0_ in eq 2 are calculated at each doping level. Therefore, all SLoT model parameters are calculated from experimental measurements. With the SLoT model, one can isolate the interrelationships between macroscopic transport properties, fundamental transport parameters (e.g., σ_0_, η(*c*), *W*_H_(*c*)), and other physical observables (e.g., interchain distances, doping level, and dopant intercalation).

Herein, we measure the charge transport properties of an *n*-dodecyl functionalized PBTTT doped with iron(III) chloride (FeCl_3_) and use the SLoT model to benchmark its charge transport properties for the first time (Figure 1a,b). Furthermore, we contextualize the PBTTT SLoT parameters by comparing with other polymer-dopant-processing systems. Finally, we expand the SLoT model and its physical significance by performing grazing incidence wide angle X-ray scattering (GIWAXS) and spectroscopic ellipsometry (SE) measurements (Figure 1c). With these techniques, we find that the localization energy is independent of paracrystalline disorder and that the carrier densities in metal-like microdomains are likely higher than that of the macroscopic average.

## Methods

PBTTT-C12 was prepared via a Migita–Kosugi–Stille (Stille) polymerization, as outlined in Note S1. The repeat unit structure was confirmed with ^1^H-NMR, as shown in Figure S1, and is consistent with previous reports.^37^ The molecular weight and dispersity (*M*_n_ = 102 kg/mol, *Đ* = 1.8, seen in Figure S2) of this polymer were estimated by gel permeation chromatography in 1,2,4-trichlorobenzene at 140 °C relative to narrow polystyrene standards. Electrochemical measurements show that the onset of electrochemical oxidation, and the redox estimated ionization potential value dictating it, is comparable to previous reports (Figure S3).^37^

PBTTT-C12 thin films (ca. 100 nm thick) were prepared by wire bar coating from chlorobenzene onto glass substrates and then thermally annealed at 180 ^°^C for 20 min, unless otherwise stated. Films were sequentially doped by dropping a FeCl_3_-acetonitrile solution onto the film and allowing the film to oxidize. After oxidation, films were rinsed with excess acetonitrile to remove excess dopant and doping byproducts and then vacuum-dried to remove solvents. Figure 1a shows the chemical structures and a representative digital photograph that shows pristine PBTTT with a subsection doped with and 50 mM FeCl_3_. Cursorily, we note that doping alters the optical absorbance of the film, which will be detailed in subsequent sections. In general, sets of films were doped and then immediately measured to mitigate the ambient environment and temporal effects. Additional procedural details and pristine PBTTT characterizations are in Note S1.

## Results and Discussion

### SLoT Modeling of PBTTT

**Figure 2:**
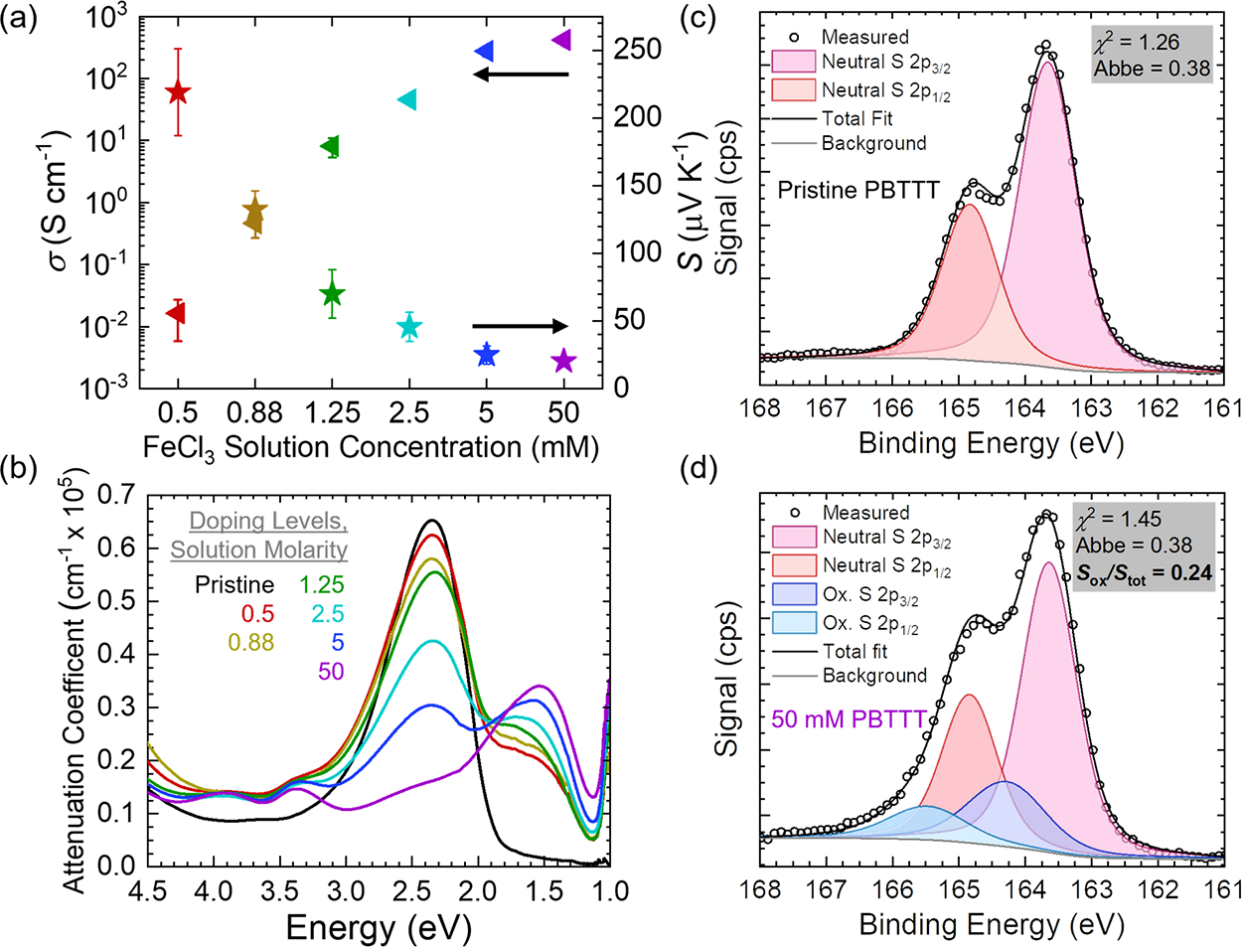
Quantifying nominal thermoelectric properties, optical properties, and extent of doping for PBTTT-C12 doped with FeCl_3_. (a) Electrical conductivity and Seebeck coefficient as a function of FeCl_3_ solution concentration. Error bars represent the sample-to-sample standard deviation. (b) UV–vis–NIR attenuation coefficient as a function of photon energy. (c) Pristine PBTTT-C12 XPS measurement and deconvolution. (d) 50 mM FeCl_3_ doped PBTTT-C12 XPS measurement and deconvolution. The low χ^2^ values, Abbe criterion, and residual signal (see Figure S6) provide a high level of confidence in these deconvolutions.

Figure 2a shows σ and *S* for PBTTT-C12 as a function of the sequential doping FeCl_3_ solution concentration. The FeCl_3_ solution concentration was systematically swept from 0.5 to 50 mM to access a wide range of extents of oxidation and thermoelectric transport properties. At 0.5 mM FeCl_3_, the PBTTT films show low σ (∼0.01 S cm^–1^) and high *S* (∼+200 μV K^–1^), indicating that these films are p-type and lightly doped. With increasing FeCl_3_ concentration, σ increases and *S* decreases, which is consistent with the expected *S* – σ anticorrelation and indicates higher carrier densities.^20^ At 50 mM, PBTTT films show a high σ (420 ± 45 S cm^–1^) and low *S* (+20 ± 2.3 μV K^–1^), consistent with several other PBTTT reports.^10,13,14,17^ We note that higher σ and lower *S* values have been reported in highly oriented thin films^11,12,18^ which can be attributed to a more ordered microstructure (improved inter-chain transport) and to a broadened electronic structure with better orbital overlap.^38,39^

To quantify the effects of FeCl_3_ doping concentration on the electronic structure and extent of doping, ultraviolet–visible–near infrared (UV–Vis–NIR) and X-ray photoelectron spectroscopy (XPS) measurements were performed. Figure 2b shows the UV–vis–NIR spectra for PBTTT films at each FeCl_3_ solution concentration. As the FeCl_3_ concentration increases, the pristine π – π* optical transition at 2.4 eV bleaches and polaronic absorbances at 1.5 eV emerge and increase, consistent with previous reports^13^ and our measured thermoelectric trends. Additionally, in the range of 3–4 eV, two peaks emerge and increase in intensity with increasing doping level. These peaks are attributed to FeCl_4_^–^ counterions, which are Coulombically associated with positive polaronic charge carriers.^15^ In Figures S5 and S6 we deconvolute these peaks and use known extinction coefficents^15,40^ to calculate the molar ratio of FeCl_4_^–^ counterions to BTTT repeat units and then the charge carrier volumetric density.^15^ At 50 mM FeCl_3_, we calculate a molar ratio of 0.80 ± 0.08 charge carriers per repeat unit. This molar ratio is equivalent to a charge carrier ratio of 0.4 (assuming two charge carriers per repeat unit)^14^ and a carrier density of 7.6 × 10^20^ carriers cm^–3^ (see PBTTT-SLoT.xlsx supporting information for calculations).^14^ The value of 0.80 ± 0.08 charge carriers per repeat unit when doped with 50 mM FeCl_3_ and measured using optical spectroscopy is consistent and within error of the XPS measurements and analysis (Figures 2c,d and S7), and the presence of FeCl_x_ counterions in the films is confirmed using survey and elemental spectra (Figure S8 and Table S1). These XPS measurements show that oxidizing PBTTT with 50 mM FeCl_3_ results in an increased signal at higher binding energies with respect to the pristine and neutral S-2p_3/2_ signal. Using previously established deconvolution procedures,^20,29,35,41−43^ we calculate that this oxidized sulfur signal is approximately 24% of the total signal in the S-2p spectra (S/S_ox_ ∼ 0.24, Figure 2d). Therefore, the XPS deconvolution indicates that approximately 1 out of every 4 sulfurs in PBTTT are oxidized, or a molar ratio of 0.96 charge carriers per repeat unit. Additionally, this maximum carrier density of 7.6 × 10^20^ carriers cm^–3^ is approximately 2× greater than those in PBTTT-F4TCNQ studies,^7,10,40^ and a PBTTT-TFSI OECT study,^14^ and BTTT copolymer doped with F4TCNQ (ranged from 3–5× 10^20^),^21^ but this carrier density is consistent with several PBTTT-FeCl_3_ studies (ranging from 5.8 × 10^20^ to 9 × 10^20^ carriers cm^–3^).^12,15,44^ While some of the differences in the calculated carrier densities could be attributed to measurement methods and assumptions (e.g., UV–Vis–NIR, XPS, AC Hall), this spread in carrier densities is also likely a function of processing conditions (e.g., dopant chemistry and reduction potential) and the resulting microstructure (vide infra).

**Figure 3:**
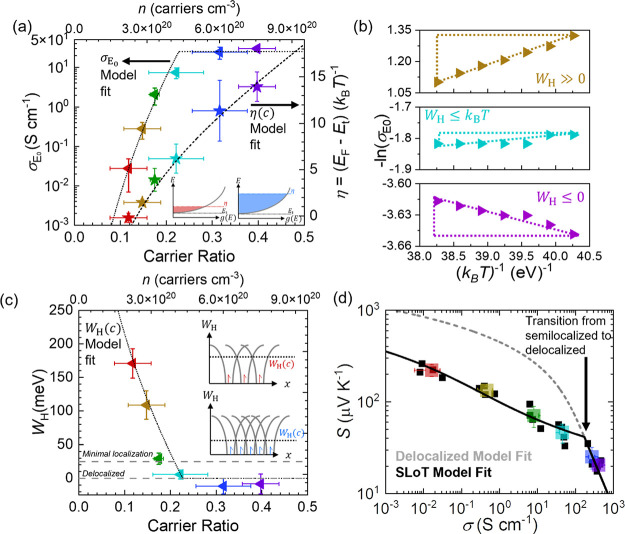
SLoT modeling of PBTTT-C12 sequentially doped with FeCl_3_. (a) SLoT model transport function prefactor and reduced Fermi energy as a function of carrier ratio and density. The inset illustrates the interpretation of increasing η values, akin to Figure 1. (b) Representative Arrhenius plots, where the slopes are equal to *W*_H_. Note that in this narrow temperature range (288–303 K), the electrical conductivity (and ergo σ_E_0__) oftentimes has a statistically significant temperature dependence while the Seebeck coefficient does not (ergo η does not observably vary). (c) Localization energy as a function of carrier ratio. The inset illustrates that localization decreases as carriers begin to spatially impinge, akin to Figure 1. Note that the carrier densities reported herein assume the carriers have a +1*e* and do not transport as a pair (i.e., bipolaron); if all charges were bipolaronic in nature, then the *n* values in (a, c) would be halved of what is presently shown. (d) *S*(σ) curve showing doping level average properties (colored squares, error bars represent sample to sample standard deviation), individual film properties (black squares), delocalized transport model (gray dashed line) and the SLoT model fit with no freely adjustable variables (black line). See Note S3 and PBTTT-SLoT.xlsx for additional details.

With these carrier ratios and thermoelectric measurements, we apply the SLoT model to gain deeper insights. Figure 3a shows σ_E_0__ and η as a function of *c*, as calculated using the thermoelectric and spectroscopic measurements from Figure 2 and eqs 1–3. σ_E_0__ is the transport function prefactor and can be calculated by dividing the measurable electrical conductivity by the calculated electrical integral (see the look-up table in PBTTT-SLoT.xlsx). Figure 3a shows that as *c* increases, σ_E_0__ exponentially increases and then plateaus near 25 S cm^–1^. Figure 3a also shows that as *c* increases, η increases, and this is interpreted as charge carriers occupying increasingly higher electronic states with respect to the transport edge. Note that when η > 0, , so η alone linearly increases σ. Lastly, we note that η increasing to a maximum of 14 (equivalent of *E*_F_ shifting by ∼0.36 eV with respect to *E*_t_ at 300 K) is consistent with the ∼0.3 eV shift intensity-weighted binding energies from XPS (Note S3 and Figure S7);^42,43^ this indicates that a linear-energy-dependent transport function (eq 1) reasonably describes the electronic structure with these chemistries and processing conditions and that the shifts in η may be used to contextualize the electronic structure.

To quantify the extent of localization, the electrical conductivity and Seebeck coefficient were measured as a function of temperature. At each temperature, *σ*_E_0__ was calculated from dividing the measured electrical conductivity by the Fermi integral value, and then *W*_H_ was calculated from the Arrhenius plot of  (Figure 3b). Figure 3b shows representative Arrhenius plots at low (0.88 mM), medium (2.5 mM), and high (50 mM) doping levels. Figure 3b,c shows that the slope and calculated *W*_H_ values start high (greater than 100 meV), decrease to the order of 25 meV (*k*_B_*T* at 300 K), and continue to decrease to negative slopes (metal-like and thermally deactivated electrical conductivities).^20,45^ These metal-like *W*_H_ values were calculated at high doping (5 and 50 mM) consistent with other reports on PBTTT.^17^ Note that although *W*_H_ can be calculated to be less than zero from temperature-dependent measurements, *W*_H_ is always modeled in eq 1 to be greater than or equal to zero because *W*_H_ captures the systematic decrease in σ due to localization effects.^20,25^ For each film and at each doping level, σ_0_ is calculated from temperature-dependent measurements, and Figure S9 shows that σ_0_ values are ca. 25 S cm^–1^, on average, and do not have a statistically significant linear dependence on η. This is consistent with the plateauing of σ_E_0__(*c*).

Finally, the *S*(σ) anticorrelation is evaluated. Figure 3d plots the *S*, σ coordinates for each film and doping level averages. The solid black curve is calculated using the SLoT model (eqs 1–3), using the experimental thermoelectric and carrier ratio measurements and their η(*c*), *W*_H_(*c*), and σ_0_ regression parameters. This SLoT model curve has no freely adjustable values and accurately captures the experimental data. Additionally, the dashed gray curve represents the delocalized *S*(σ) anticorrelation (i.e., Kang–Snyder *s* = 1) which assumes that σ_E_0__ = σ_0_ at all doping levels and that σ_E_0__ (and ergo *W*_H_) does not change as a function of doping level.^23^ Notably, the SLoT model and delocalized models become colinear when *W*_H_ ≲ *k*_B_*T* and capture the data points that have little to no thermal activation at high doping levels. We note that the SLoT fit is calculated with no freely adjustable parameters in Figure 3d, and this fit is remarkably consistent with the SLoT fits previously predicted using previous literature data sets and an adjustable *W*_H_(*c*) relationship (Figure S10).^14,17,20^ This consistency further affirms the use of the SLoT model to predict transport parameters from *S*(σ) data when temperature- and/or carrier-dependent data are not available.

### Developing Microstructural-Charge Transport Relationships

The SLoT model becomes more useful when coupled with additional characterization techniques. At minimum, the SLoT model requires temperature-dependent thermoelectric and carrier density measurements, but these measurements do not quantify how the atomic structure and/or microstructure affect the resulting observables. Therefore, we now examine how GIWAXS furthers the utility of the SLoT model for this PBTTT-FeCl_3_ system.

**Figure 4:**
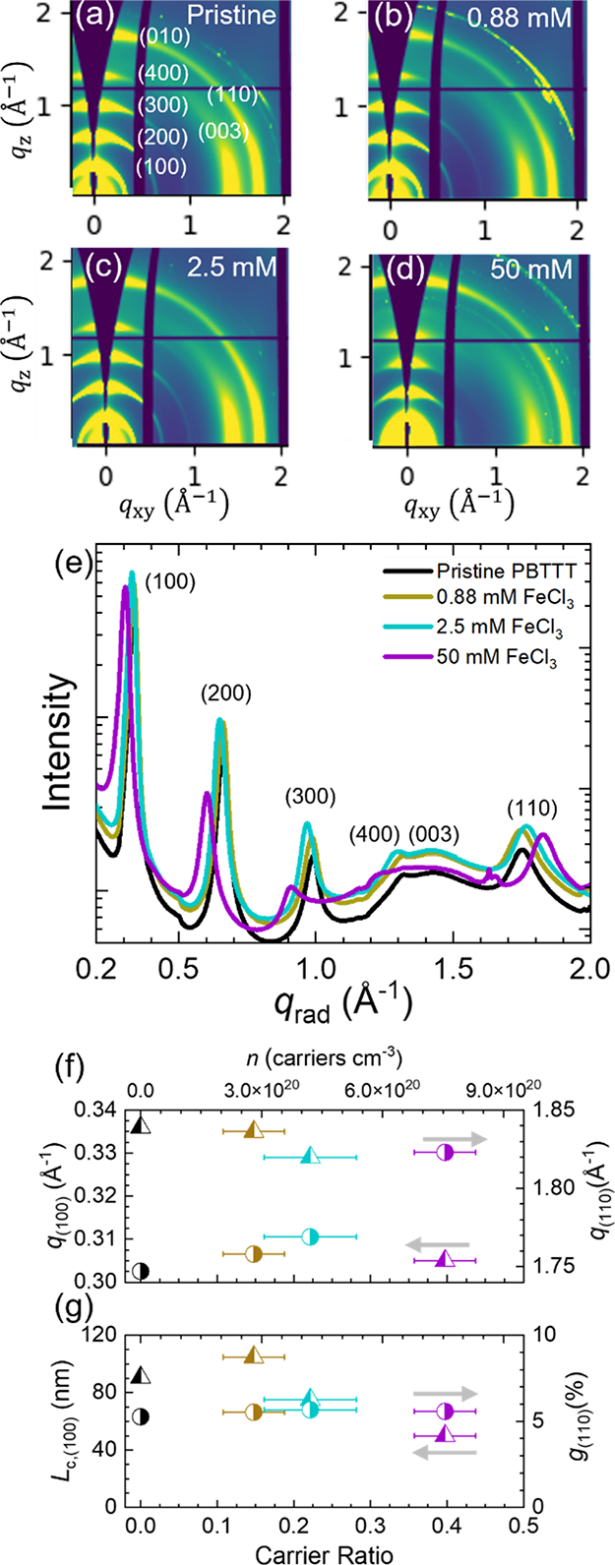
PBTTT-FeCl_3_ GIWAXS measurements and analysis. Representative diffractograms for (a) pristine, (b) 0.88 mM FeCl_3_ doped, (c) 2.5 mM FeCl_3_ doped, and (d) 50 mM doped PBTTT films. Pristine diffractogram shows annotated indices. (e) Radially integrated linecuts. (f) Lamellar (100) and π – π *q* values. (g) Coherence length and paracrystallinity. Note that in (f) and (g), the top and bottom *x*-axes are the same for both plots and that the arrows correspond to the *y*-axis for each data series. Explicitly, the triangle data points correspond to the left *y*-axes while the circles correspond to the right *y*-axes.

Figure 4a–d shows representative two-dimensional diffractograms for pristine, 0.88, 2.5, and 50 mM doped films. These diffractograms show a high extent of ordering for a conjugated polymer system, with lamellar (*h*00) peaks up to a (400) reflection, consistent with previous reports on PBTTT.^8,15^ Furthermore, these diffractograms show that the crystallite lamellar stacking direction is predominantly along the out-of-plane direction (*q_z_* axis), and the (003) intrachain spacing is predominantly along the nearly-in-plane (*q_xy_*) axis. Additionally, there is a distribution of (110) interchain π – π stacks, with a bimodal predominance near the two poles at all doping conditions. These orientations indicate that these PBTTT films are preferentially ordered edge-on with respect to the substrate; however, we note that this extent of edge-on is qualitatively weaker than that reported in other studies and may be because these films are thicker (hundreds of nanometers) compared to those commonly used in other studies (tens to hundreds of nanometer).^15^

To gain deeper insight into these diffractograms, we analyzed radially integrated profiles (Figure 4e). These profiles show that as the extent of doping increases, the lamellar peaks shift to small *q* values, indicating an expansion in real space from 18.7 to 20.6 Å, as shown in Figure 4f. Concomitantly, the π – π stacks contract in real space from 3.58 to 3.45 Å, which suggests dopant intercalation in the side chain region, consistent with previous reports.^10,15^ The PBTTT-C12 reported here has smaller lamellar spacings compared to PBTTT-C14 (20 Å pristine, 26 Å FeCl_3_ doped),^15^ as expected, but has notably tighter π – π stacks compared to previous PBTTT-C_x_ reports (∼3.7 Å pristine, ∼3.55 Å doped).^15,17^ These tighter π – π stacks may be afforded by the smaller side chains and likely improve charge transport within crystalline domains. Furthermore, the quality of the crystalline domain can be evaluated using the paracrystallinity (*g*) and the coherence length (*L*_c_).^46−48^ Detailed notes on the interpretation and calculations of *g* and *L*_c_ are found in the Supporting Information (Note S4, Figures S11–S12, and Table S2), and here, we highlight two key findings. First, Figure 4g shows that *g* = 5.5%, on average, for this PBTTT-C12/FeCl_3_ system in the (110) direction. This is a remarkably low level of paracrystalline disorder for a conjugated polymer (generally ∼7–20%).^49^ Therefore, we believe that the ordered domains are exceptionally well ordered and tightly packed, but this amount of order does not provide insight on percolation pathways between spatially separated domains. Second, we note that the amount of paracrystallinity (i.e., structural disorder) in the (110) direction varies slightly (∼10%) as a function of doping level; but, the coherence length (i.e., crystallite size) in the (100) direction significantly decreases (−44%) with increasing doping level (Figure 4g). This indicates that FeCl_3_ doping substantially decreases the quality of the ordering of the electrically insulating alkyl region, but doping does not substantially affect the quality of the ordering of the electrically conductive π – π stacking direction.

The fact that *g* is independent of doping level is quite notable because previous reports have linked (110) paracrystallinity to the broadening of the density of electronic states and the creation of trap and/or localized electronic states; these trap states have energetic breadths and field-effect thermal activation energies on the order of ca. 100 meV in the pristine polymer.^28,46−50^ Similarly, in the dilute carrier limit (*c*∼0.1), the localization energy (*W*_H_) is within a factor of 2× as predicted using paracrystallinity models. Although *g* remains effectively constant with respect to the doping level, we observe that *W*_H_ decreases significantly with increasing doping level. We hypothesize that this apparent discrepancy between *g* and *W*_H_ at higher doping levels is because the reduced Fermi energy levels (and transport properties) probed are not at the same energy levels where there is energetic disorder due to paracrystallinity. This hypothesis is reinforced by the fact that when PBTTT is “doped” using a field effect method (i.e., controlling the applied biases), PBTTT oftentimes obtains Seebeck coefficients on the order 700–1000 μV K^–1^,^51^ which is akin to an η of −7 (∼0.2 eV below *E*_t_ and ∼0.55 eV below 50 mM FeCl_3_ doped PBTTT herein). Therefore, this study suggests that the (110) paracrystallinity and structural disorder is not a significant contributing factor to *W*_H_ at these degenerate doping levels, and we hypothesize the physical mechanisms that limit *W*_H_ in chemically doped semiconducting polymers are likely the electrostatic attraction of polaronic charge carriers to counteranions, polarization of the local bond order, and the spatial percolation of charge carriers on larger length scales (>∼10 nm).^20,25,27^

### Quantifying Localization and Charge Transport Using Spectroscopic Ellipsometry

The SLoT model uses macroscopic thermoelectric measurements to define transport parameters that represent an appropriately weighted average of the microscopic ensemble. Though SLoT allows us to model the macroscopic average, conjugated polymers are known to be inhomogeneous. Explicitly, the macroscopic σ is a single value, but it is known that there are regions within the polymer that have larger σ values that are likely electrically insulated (to some extent) by regions with smaller σ values.^30,52,53^ This inhomogeneity has been used to improve charge transport and thermoelectric performance; for example, Ma et al. recently demonstrated that higher thermoelectric power factors are obtained by preferentially doping the ordered regions.^53^ Although *W*_H_ is a useful tool to capture the macroscopic effects of localization, and GIWAXS is a useful tool to quantify the quality and spacing or molecular packing within crystalline domains, these approaches do not provide information on how the ordered domains are distributed and/or percolated throughout the film and their microscopic transport properties. Measurement techniques, such as atomic force microscopy,^52^ scanning kelvin probe microscopy,^54^ atom probe tomography,^55^ and transmission electron microscopy,^56^ can have sufficient spatial resolution to quantify inhomogeneous spatial distributions, but these techniques do not necessarily provide information on the microscopic transport properties. In contrast, there are reports that use thermal^57,58^ and dielectric properties^59−62^ to contextualize the effects of percolation and effective medium inhomogeneity on the resulting σ(*n*, *T*) and *S*(*n*, *T*) properties.^63^ Ultimately, we posit that SE measurements are useful for characterizing inhomogeneous materials with microscopic regions of varying transport properties.

Here, we explore the use of SE to quantify the complex dielectric function in PBTTT, model the dielectric-like and metal-like contributions to the observable optical properties, and relate these contributions to the measurable thermoelectric properties and SLoT parameters. The complex dielectric function is expressed as
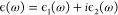
4where ϵ_1_(ω) is the real component, and ϵ_2_(ω) is the imaginary component.^64^ The complex dielectric function can link measurable optical properties, such as reflectivity, to the calculable material parameters, such as carrier densities. Additionally, ϵ_1_(ω) and ϵ_2_(ω) capture various physical processes such as charge polarization, interband optical transitions, and Drude-like charge transport.^64,65^

**Figure 5:**
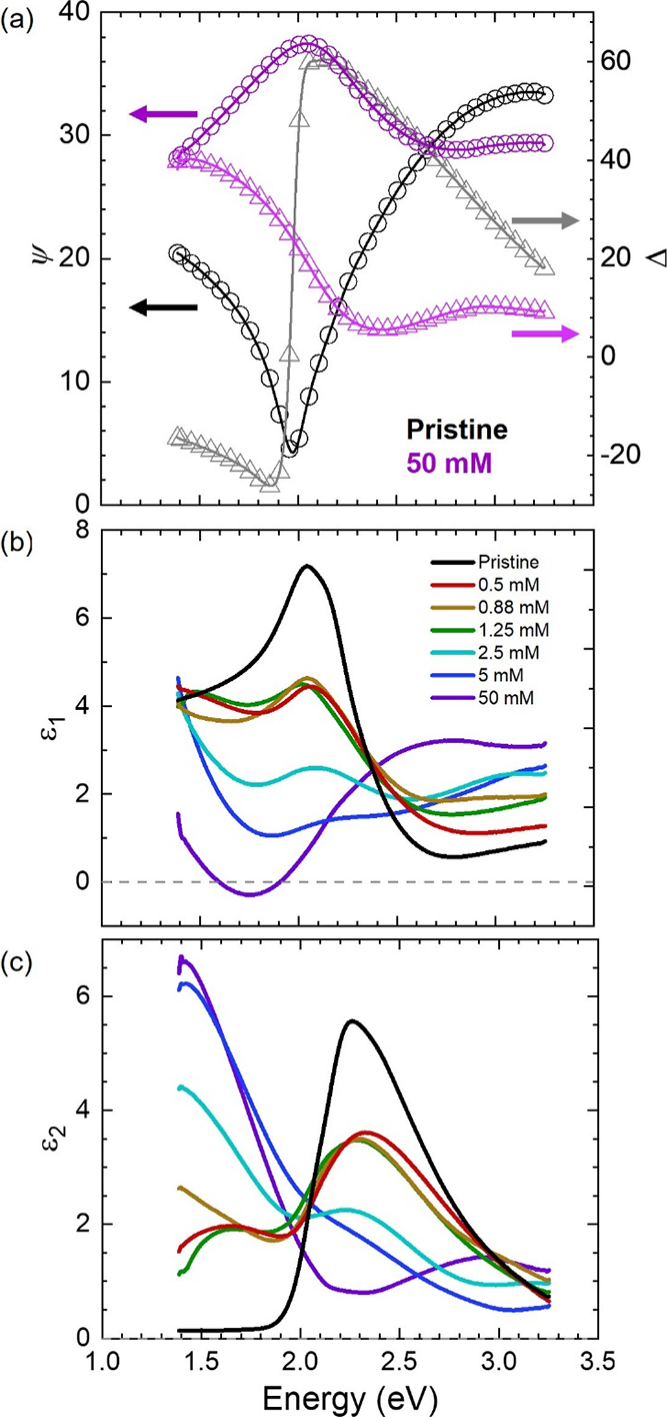
PBTTT-FeCl_3_ spectroscopic ellipsometry measurements, B-spline fits, and complex dielectric function calculations. (a) Representative ψ and Δ measurements for pristine and 50 mM FeCl_3_-doped PBTTT films with comparable thickness (∼180 nm) on glass substrates. One out of every five measured data points are shown for clarity, and lines represent the B-spline fitting, calculated using known substrate properties, known film thickness, and assumed Kramers–Kronig consistency. (b) Real component (ϵ_1_) of the complex dielectric function. (c) Imaginary component (ϵ_2_) of the complex dielectric function.

SE measures the change in the ratio of the polarized light intensities (tan(ψ)) and phases (Δ) upon reflection,^64,66,67^ and these changes in ψ and Δ are related to ϵ_1_(ω) and ϵ_2_(ω) through an optical model. Figure 5a shows representative ψ and Δ values for pristine PBTTT and 50 mM FeCl_3_ doped PBTTT with comparable thicknesses, and Figure S13 shows ψ and Δ at each doping level. Clearly, the measurable ψ and Δ change due to doping, consistent with the prior characterizations, and this suggests that ϵ_1_(ω) and ϵ_2_(ω) also change. To quantify ϵ_1_(ω) and ϵ_2_(ω) in the PBTTT film, an empirical optical model was developed to fit the changes in ψ and Δ and extract the film’s dielectric function (Note S5 details the optical model). Figure 5a shows the B-Spline fits from the optical model agree well with the measured values, indicating that the ϵ_1_(ω) and ϵ_2_(ω) values calculated with the optical model for the PBTTT films are consistent with the experimental measurements.

Figure 5b,c plots the calculated ϵ_1_(ω) and ϵ_2_(ω) as a function of doping level and photon energy. ϵ_1_ is associated with the ideal polarization of the film without losses, and ϵ_2_ is associated with lossy processes such as heat generation, interband absorption, and free charge carrier absorption.^64,65^ The values for ϵ_1_(ω) and ϵ_2_(ω) (and their corresponding complex index of refraction values, *n*∼ and *k*∼, Figure S14) are reasonable and consistent with previous measurements on doped and pristine conjugated polymer films.^66−68^ These consistencies give us a high level of confidence in the ϵ_1_(ω) and ϵ_2_(ω) calculations.

Most notable is that ϵ_1_ in the 50 mM PBTTT shows regimes where ϵ_1_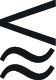 0 (Figure 5b). Typically, metals have negative ϵ_1_ values due to free carrier attenuation at frequencies less than the plasma frequency and a positive ϵ_1_ at frequencies greater than the plasma frequency due to polarization.^64^ In contrast, Lorentz-like oscillators (e.g., band gap transitions) typically exhibit ϵ_1_ > 0. Because 50 mM doped PBTTT exhibits both positive and negative ϵ_1_ values, it is likely that a combination of free-electron and optical transitions is present, consistent with the UV–Vis–NIR measurements and thermoelectric measurements. This is qualitatively represented by the cartoon illustration in Figure 1c, where the SE is probing and measuring transitions in multiple spatial regimes. Furthermore, previous reports have also observed similar ϵ_1_ behavior with PEDOT:PSS and PEDOT:Tos as a function of processing conditions and doping level (where ϵ_1_ changes sign multiple times),^69−72^ but these studies did not systematically vary the doping level from the pristine state to a fully doped state. Finally, the optical transitions and ϵ_2_ values in Figure 5 are in good agreement between and the transmission attenuation in Figure 2. Both analyses show that absorption shifts to the infrared region and the visible region is increasingly bleached with increasing doping level.

We now turn to deconvoluting ϵ(ω) to better quantify the physical phenomena responsible for the optical transitions. By deconvoluting ϵ(ω), we obtain fundamental transport properties such as the carrier density. Here, we deconvolute ϵ(ω) using the simplest peak deconvolution models that account for polaronic transitions, free charge carriers, and π – π* transitions; however, we note that more complicated models exist that account for specific microgeometries (e.g., core–shell, slabs, etc.), in- and out-of-plane contributions (i.e., ordinary, and extraordinary), and grating.^65,73,74^

Overall, ϵ(ω) has the functional form of
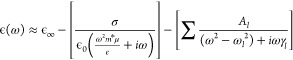
5ϵ_∞_ is the dielectric constant at approaching infinitely high frequencies, ϵ_0_ is the permittivity of free space, ω is the angular frequency, *A_l_* is the *l*-th Lorentzian absorption amplitude, ω_*l*_ is the resonant frequency of the *l*-th absorber, and γ_*l*_ is the absorption broadening. The first bracketed term accounts for Drude-like free carrier contribution, and the second bracketed term accounts for sum of *l*-number of oscillator transitions, such as π – π* optical, polaronic, and vibrational transitions.^73,74^ The second bracketed term is written in the form of a Lorentzian oscillator, but depending on the exact mechanism, the broadening of the Lorentz line shape may be more appropriately modeled using a single Gaussian, multiple Gaussians, Tauc–Lorentz, or Voigt distributions, to name a few.^74^ By deconvoluting ϵ_1_ and ϵ_2_ at each doping level, the contribution of each oscillator and free-electron contribution can be systematically quantified.

**Figure 6:**
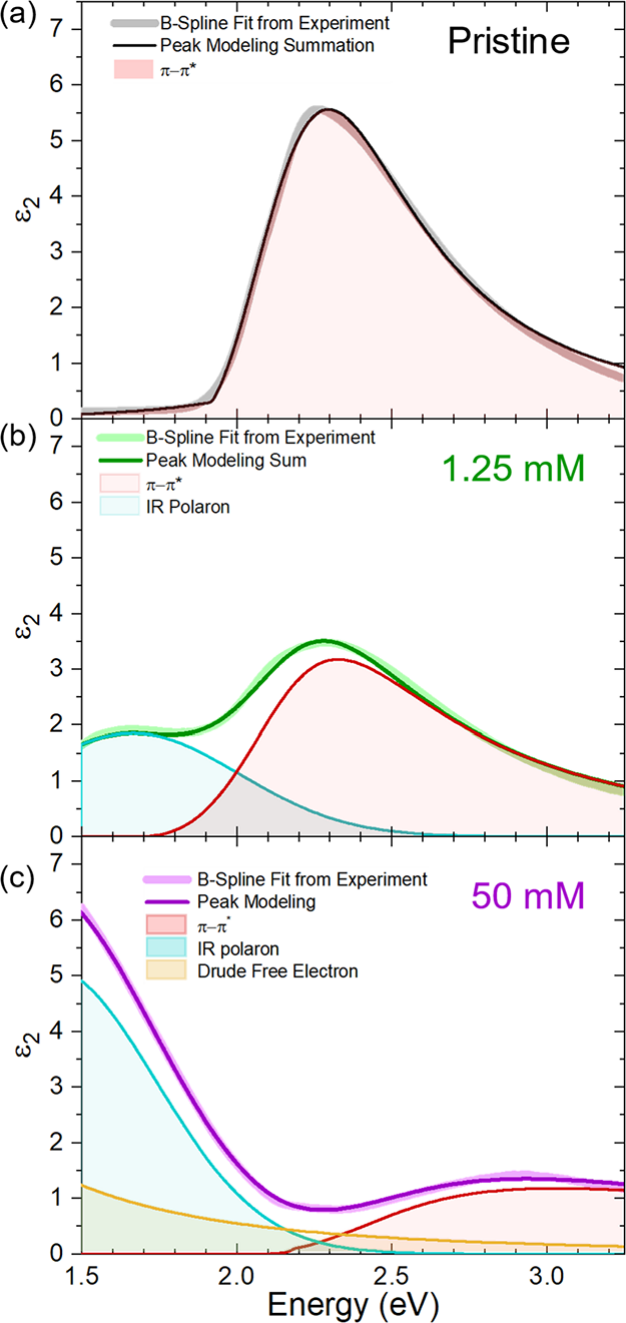
Representative SE deconvolutions as a function for PBTTT-FeCl_3_ doping level. (a) Pristine PBTTT, modeled using only a Cody-Lorenz oscillator for the π – π* band gap transition. (b) PBTTT doped with 1.25 mM FeCl_3_, modeled using a compared π – π* band gap transition and a Gaussian polaronic absorption. (c) PBTTT doped with 50 mM FeCl_3_, modeled using a comparable −π* optical transition and polaronic absorption as well as a Drude free electron contribution. Additional deconvolution notes and methodologies are found in Note S5.

Figure 6 shows a representative set of spectroscopic ellipsometry deconvolutions, which isolate the physical contributions to the dielectric loss function. Figure 6a shows the deconvolution for pristine PBTTT. The B-spline fit is well modeled using a single Cody–Lorentz peak, which captures the optical band gap, the π – π* transition. While Lorentzian peaks are symmetrical about the harmonic frequency, Cody–Lorentzian peaks are asymmetric about the harmonic frequency and account for more transitions above the harmonic frequency and a steeper cut-off for transitions below the harmonic frequency.^75^ Although multiple peaks could be used to deconvolute the π – π* transition,^76^ this becomes increasingly cumbersome when deconvoluting the doped films as there are more free variables. Using the peak deconvolution settings from the pristine PBTTT, we then turn to quantifying the polaronic oscillator contribution. Figure 6b shows the deconvolution for 1.25 mM FeCl_3_ doped PBTTT. At 1.25 mM, σ, *n*, and μ values are likely too low to significantly contribute to the dielectric function in the UV–Vis–NIR region, but there is a polaronic peak near 1.5 eV, akin to Figure 2. This peak is well modeled using a simple Gaussian peak, like previous reports.^76^ Finally, Figure 6c shows 50 mM FeCl_3_ doped PBTTT. Using the incremental change in the dielectric function at each doping level (Figure 6), we can begin to isolate the free electron contribution to ϵ_2_. Notably, Figure S15 shows that the 50 mM dielectric function cannot be as well modeled using a polaronic and π – π* oscillators alone; therefore, we conclude that the Drude free electron contribution is likely necessary. Additionally, this Drude-like contribution is needed to explain the slopes and curvatures in the mid-infrared (MIR) as shown in Figure S6. We note that the Drude contribution in the MIR is convoluted with polaronic absorbances (likely a combination of polaron and bipolaron contributions), and therefore, this Drude contribution may be responsible for asymmetric peaks that decreases with increasing energy. Future SE measurements deeper in the MIR and on additional highly electrically conductive polymers will increase the certainty of the Drude contribution in spatially inhomogeneous polymers.

This Drude contribution at 50 mM is modeled by using *n* = 6 × 10^21^ carriers cm^–3^ and σ = 2200 S cm^–1^ (Figure 6c). Although this Drude contribution is consistent with spectroscopic ellipsometry data, it is seemingly inconsistent with the thermoelectric transport data which shows *n* = 7.6 × 10^20^ carriers cm^–3^ and σ = 420 S cm^–1^ (Figures 2 and 3). Interestingly however, we note that this ellipsometry carrier density is comparable to the carrier density recently reported using an AC Hall technique.^44^ We believe that these substantial differences in transport properties is likely because the ellipsometry measurements are probing optical oscillators which are on smaller length scales compared to the macroscopic thermoelectric transport (Figure 1c). It is likely that the carrier densities and electrical conductivities calculated using the Drude component from ellipsometry deconvolutions are a more accurate representation for the electrically conductive domains (likely crystalline regions of the π – π stacks), whereas the carrier densities and electrical conductivities in Figures 2 and 3 are better thought as bulk averages, which are systematically lowered by less ordered domains and insulating side chains. We note that PBTTT is ∼50% insulating side chain by molecular weight, and we recently demonstrated that removing the side chains in dioxythiophene copolymers increases the measure σ by ∼10× and *n* by ∼2×.^29,77^ Furthermore, the Drude electrical conductivity modeled using ellipsometry is consistent with several reports that show σ in PBTTT is >10^3^ S cm^–1^ when highly ordered.^12,18,19^ This ellipsometry analysis suggests that highly electrically conductive PBTTT on the macroscale is afforded by a percolated series of highly ordered and Drude-like domains, and less electrically conductive PBTTT on the macroscale is due to highly ordered Drude-like domains being electrically isolated from one another. This percolation picture is akin with previous ellipsometry-charge transport reports that compared the differences in ϵ and σ as a function of evaporated Au film thickness,^61^ atomic layer deposited Pt/Ru/Pd film thickness,^62^ and the percolation of Ag nanoparticles in a strained elastomer.^60^ Ultimately, this analysis shows that spectroscopic ellipsometry could be useful for complimenting structural and thermoelectric measurements and provides a more holistic picture for charge transport.

## Conclusions

The charge transport properties of chemically doped semiconducting polymers are difficult to holistically understand because spatial inhomogeneity leads to charge transport properties that vary significantly as functions of doping level and spatial coordinate. In this PBTTT-FeCl_3_ study, we used the SLoT model to quantify fundamental transport parameters, such as σ_0_, *W*_H_(*c*), and η(*c*), which can be used to robustly compare and design polymer-dopant-processing systems. Using GIWAXS measurements, we conjecture that PBTTT’s highly conductive transport parameters are likely because of the smaller π – π stacking distances that decrease with the increasing doping level and have low levels of paracrystalline disorder. Using SE measurements and deconvolution, we quantify how the complex dielectric function varies with doping and model the Drude contribution to optical properties. We find that these Drude carrier densities and electrical conductivities are larger compared to the SLoT parameters, and we hypothesize that this could be because only the most electrically conductive and metal-like domains will have Drude like optical signatures, while the SLoT parameters weighted by the electrically insulating domains and are more indicative of macroscopic averages.

Moving forward, this study serves two primary purposes. First, we contextualized the SLoT transport parameters for PBTTT-FeCl_3_ against other PBTTT studies and semiconducting polymer systems. This benchmark can be used moving forward to better quantify, understand, and compare other polymer-dopant-processing systems. Second, we showed how advanced scattering and spectroscopic measurements can be used in conjunction with SLoT to better understand transport properties. With additional SLoT, GIWAXS, and SE measurements, we can better quantify to what extent microstructure and inhomogeneity affect the resulting transport properties.

## Supporting Information Available

SLoT measurements, calculations, and look-up table (XLSX)
Additional characterization procedures and measurements (PDF)
