# Supplementary material for: Quantifying Charge Carrier Localization in PBTTT Using Thermoelectric and Spectroscopic Techniques
Source: J Phys Chem C Nanomater Interfaces. 2023 Jun 14;127(25):12206–17. doi: 10.1021/acs.jpcc.3c01152 (PMC10320779; doi:10.1021/acs.jpcc.3c01152)
Supplement: Supplementary file 2 — jp3c01152_si_002.pdf [file jp3c01152_si_002.pdf]

Quantifying Charge Carrier Localization in PBTTT using Thermoelectric and Spectroscopic Techniques

Shawn A. Gregory,[1] Amalie Atassi,[1] James F. Ponder Jr.,[2] Guillaume Freychet,[3] Gregory M. Su,[4,5] John R. Reynolds,[1,6] Mark D. Losego,[1] Shannon K. Yee[2*]

1 School of Materials Science and Engineering, Georgia Institute of Technology, Atlanta, GA, 30332, USA.

2 George W. Woodruff School of Mechanical Engineering, Georgia Institute of Technology, Atlanta, GA, 30332, USA

3 NSLS-II, Brookhaven National Laboratory, Upton, New York, 11973, United States

4 Advanced Light Source, Lawrence Berkeley National Laboratory, Berkeley, California, 94720, United States

5 Materials Sciences Division, Lawrence Berkeley National Laboratory, Berkeley, California, 94720, United States

6 School of Chemistry and Biochemistry, Georgia Institute of Technology, Atlanta, GA, 30332, USA.

* Corresponding author: shannon.yee@me.gatech.edu

Contents

Note S1: Experimental Procedures and Pristine PBTTT Characterization 3

Scheme S1: Synthesis of PBTTT-C12 3

Figure S1: PBTTT ^{1}H-NMR spectrum in TCE-D_2 at 100 °C. 7

Figure S2: PBTTT GPC chromatograph in TCB at 140 °C. 8

Figure S3: Electrochemistry of a PBTTT film on a glassy carbon button electrode in $TBAPF_6$/PC. (a) CV. (b) DPV. 9

Figure S4: DSC measurements of PBTTT. (a) as cast and cycled from -50 C to 275 °C. (b) re-solvated and remeasured cycling from 0 to 300 °C. (c) Thermally annealed akin to the wire-bar films and cycled -50 to 290 °C. 10

Note S2: UV-Vis-NIR Peak Deconvolution for Carrier Concentration Calculations 11

Figure S5: UV-Vis-NIR spectra used for $FeCl_4^-$ peak deconvolution 12

Figure S6: Deconvolutions used to calculate the attenuation coefficient peak heights and then the FeCl4- concentrations. (a) 50 mM (b) 5 mM (c) 2.5 mM (d) 1.25 mM (e) 0.88 mM (f) 0.5 mM. 13

Note S3: SLoT modeling notes 14

Figure S7: X-ray photo electron spectroscopy measurements and deconvolutions for representative doping levels. (a,b,e,f) measured and deconvoluted spectrograms. (c,d,g,h) calculated fitting errors as calculated from residuals/ envelope x 100% 16

Figure S8: X-ray photo electron spectroscopy measurements for (a) chlorine and (b) iron. Vertical axes are offset to facilitate comparisons between spectra. 17

Table S1: XPS Survey Atomic Abundances 17

Figure S9: $\boldsymbol{\sigma 0}$ evaluations. (a) $\boldsymbol{\sigma 0(\eta)}$. (b) Distribution of $\boldsymbol{\sigma 0}$. 18

Figure S10: Comparison of PBTTT SLoT predictions with SLoT calculations. 18

Note S4: GIWAXS 19

Figure S11: Representative GIWAXS deconvolution. Raw data (grey curves) was fitted by a series of Gaussian-Lorentzian curves (colored and shaded areas). Black curves represent the baseline and total peak fits. 20

Figure S12: GIWAXS Analysis. (a) Representative plot showing that the peak broadens with respect to the peak reflection maximum location ($\boldsymbol{q0}$). (b) $\boldsymbol{\Delta q}$ as a function of the (h00) reflection. 20

Table S2: Coherence Length tabulations 21

Note S5: Spectroscopic Ellipsometry and Attenuated Total Reflectance Infrared Absorbance Measurements 22

Figure S13: $\boldsymbol{\psi}$ and $\boldsymbol{\Delta}$ measurements. (a) Pristine PBTTT. (b) 0.5 mM FeCl3 doped. (c) 0.88 mM. (d) 1.25 mM. (e) 2.5 mM. (f) 5 mM. (g) 50 mM. 25

Figure S14: $\boldsymbol{n}$, $\boldsymbol{k}$, $\boldsymbol{\alpha}$ optical constants from SE. (a) Real part of the complex refractive index. (b) Imaginary part of the complex refractive index. (c) Absorption coefficient. 26

Figure S15: Fitting 50 mM PBTTT SE measurements with and without Drude free electron contributions. 27

Figure S16: ATR-FTIR measurements and optical constants. (a) Approximated attenuation coefficients, calculated from the measured absorbance normalized by film thickness. (b) Approximated imaginary part of the refractive index. Note that these values are approximates, as an ATR technique was employed- not transmittance complemented with reflectance nor ellipsometry. 28

References 29

Note S1: Experimental Procedures and Pristine PBTTT Characterization

PBTTT Synthesis: 5,5′-Dibromo-4,4′-didodecyl-2,2′-bithiophene (97%), 2,5-bis(trimethylstannyl)-thieno[3,2-b]thiophene (97%), tris(o-methoxyphenyl)phosphine [(*o*-MeOPh)$_3$P), 96%], 2-(tributylstannyl)thiophene (97%), and 2-bromothiophene (98%) were obtained from Sigma and used as received. Pd_2dba_3-$CHCl_3$ was prepared by recrystallizing commercially purchased Pd_2dba_3 using a previously described method.[1]

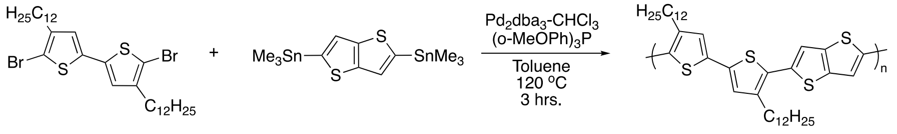

Scheme S1: Synthesis of PBTTT-C12

5,5′-Dibromo-4,4′-didodecyl-2,2′-bithiophene (371.6 mg, 0.56 mmol, 1 eq.), 2,5-bis(trimethylstannyl)-thieno[3,2-b]thiophene (262.4 mg, 0.56 mmol, 1 eq.), Pd_2dba_3-$CHCl_3$ adduct (10.7 mg, 0.01 mmol, 0.02 eq.), and (*o*-MeOPh)$_3$P (15.6 mg, 0.04 mmol, 0.08 eq.) were added to a 20 mL microwave vial along with a stir bar. Degassed toluene (5.6 mL) was added to the vial and the vial was purged with Ar before sealing. The reaction mixture was heated to ~120 °C for 3 hours. The polymer was then end-capped by first adding 0.1 mL of 2-(tributylstannyl)thiophene and mixing at temperature for 1 hour, followed by adding 0.2 mL of 2-bromothiophene and mixing at temperature for 1 hour. The solution was then cooled to ambient temperature, diluted with chlorobenzene, warmed to dissolve the contents, and precipitated into methanol. The precipitate was filtered into a soxhlet thimble and purified via soxhlet washing. The polymer was washed with methanol, acetone, ethyl acetate, hexane, tetrahydrofuran, chloroform, and then dissolved into hot chlorobenzene. The polymer solution was reprecipitated into methanol, filtered, and dried under vacuum to yield 343.5 mg (96%) of PBTTT as a hard black/purple solid. ^{1}H-NMR (400 MHz, $C_2D_2Cl_4$, 100 °C, 32 scans) δ 7.35 (s, 2H), 7.11 (s, 2H), 2.88 (br, 4H), 1.79 (br, 4H), 1.37 (br, 36H), 0.94 (br, 6H). NMR spectrum consistent with previous reports.[2] M_n = 102 kg/mol (~ 160 repeat units), $Đ$ = 1.8, vs. PS in TCB at 140 °C vs. PS standards.

NMR and GPC: The NMR spectrum (collected over 32 scans) was collected on a Bruker Avance III 400 instrument using 1,1,2,2-tetrachloroethane-D_2 as the solvent at a temperature of 100 °C.

Molecular weight information was collected on a Tosoh ECOSEC High-Temperature GPC using 1,2,4-trichlorobenzene as the elutent at 140 °C. Results are relative to narrow molecular weight polystyrene standards. As the PBTTT sample dissolves well when heated, as seen by a light orange solution compared to the arrogated purple solution at RT, and the GPC trace show no unusual peak shape, we expect the high M_n measured is accurate and not the result of arrogation.

Electrochemistry: Electrochemical measurements (CV and DPV) were performed using a model 273A EG&G PAR potentiostat/galvanostatic controlled by CorrWare. The electrical potential in the three-electrode cell setup was measured using a Ag/AgCl reference electrode (potentials reported relative to the ferrocene ferrocenium redox couple, as measured using this setup). $TBAPF_6$ (recrystallized from ethanol) was used as the supporting electrolyte (0.5 M in propylene carbonate) and a platinum flag was used as the counter electrode. Films were prepared by drop casting 1 µL of a 8 mg/mL solution of PBTTT in hot chlorobenzene onto a glassy carbon button electrode (area = 0.07 cm^2).

Ionization potentials were measured by differential pulse voltammetry (DPV) on a freshly processed film, giving an onset at +0.19 V versus ferrocene/ferrocenium which correlates to an IP of 5.29 eV. This calculation assumes that ferrocene has an onset of oxidation of -5.1 eV,[3] and that DPV measured and calculated values are commensurate to solid-state properties.[4]

Differential Scanning Calorimetry: DSC measurements were performed on TA Instruments DSC250 Differential Scanning Calorimeter using Tzero pans and a measurement range from to using a rate of 10 °C /min. Two samples of PBTTT-C12 of approximately 4 mg each were prepared for calorimetry by first dissolving the polymer in chlorobenzene at a concentration of 10 mg/mL, heating the solution for 1 hour at 80 °C, then drop-casting the solution onto clean glass substrates with a substrate temperature of 80 °C.

One of these 4 mg samples, denoted as the "as cast" sample, was measured as a reference for the PBTTT-C12 polymer (see **Figure S4a**). The as-cast sample shows two well-defined endotherm melting peaks with onset temperatures of 170 °C and 260 °C and complementary exotherms with onset temperatures of 140 °C and 260 °C. These thermal transitions are often ascribed to side-chain and backbone chain melting, respectively. Depending on the literature reference, these strong endotherms for PBTTT-C12 can be consistent[2, 5] or inconsistent[2, 5, 6] with previous reports on PBTTT derivatives (including PBTTT-C12).

After this first experiment was conducted, denoted as "exp.1," the sample was subsequently re-solvated, re-processed as described earlier, and re-measured (see "exp. 2") to determine if the DSC measurement altered the chemical structure or degraded the material. The thermograms in **Figure S4a** and **Figure S4b** are nearly identical, indicating that this DSC procedure does not affect the inherent chemical structure of the PBTTT-C12 reported herein.

The second drop-casted sample was thermally annealed similar to wire-bar films by placing the drop-casted films onto a hot plate at 180 °C for 20 minutes under dark and ambient atmosphere. This thermally annealed sample was measured as a reference for quantifying the effects of thermal annealing on polymer structure, used for the report thermoelectric, GIWAXS, and SE measurements. Relative to the as-cast samples, **Figure S4c** shows that thermally annealing PBTTT-C12 decreases the enthalpic peak area and intensity with respect to the as cast films. This decrease in enthalpic peak area suggests that thermal annealing PBTTT-C12 inhibits thermal transitions for the PBTTT-C12 reported in this study. This enthalpic inhibition indicates that there is less crystalline material to melt at these temperatures. Although we cannot robustly claim to what extent which mechanism is responsible for this decrease in DSC endotherms after annealing, we note that the C-1s and S-2p XPS spectra for PBTTT-C12, after wire-bar coating and annealing, is akin to many other alkyl thiophenes; therefore, XPS data suggests that this annealing process does not significantly oxidize or chemically degrade the PBTTT-C12 surface. Additionally, we note that GIWAXS diffractograms for PBTTT-C12, after wire-bar coating and annealing, show strong diffraction peaks (multiple order reflections and low paracrystallinity values); therefore, GIWAXS data suggests that this annealing process does not completely remove all ordering in the PBTTT-C12 reported herein. We note that there are several studies that report on PBTTT derivatives (including -C12) that have small and diffuse DSC melting endotherms but rather prominent GIWAXS peak signal.[2, 5-7]

Film Preparation: A 10 mg/mL solution of PBTTT in chlorobenzene was heated to 80 °C for one hour. Before coating, Au contact pads (150 nm thick, 5 mm apart, arranged in a van der Pauw geometry) were evaporated onto cleaned microscope slides with a 5 nm titanium adhesion layer underneath. Glass slides were cleaned before Au deposition and before film coating by sonicating in acetone (5 min), followed by isopropyl alcohol (5 min), drying with N_2 and treating with ultraviolet–ozone (5 min). Then, the prepared solution (60 μl) was cast onto a 7.6 cm × 2.5 cm glass slide and wire bar coated at approximately 180 mm s^{-1} using a substrate temperature of 80 °C on a K Control Coater with a close-wound wire diameter of 0.3 mm. After coating, thin-film samples were prepared by carefully cutting the 7.6 cm × 2.5 cm film into 1 cm × 1 cm squares. After film preparation, PBTTT films were thermally annealed at *ca.* 180 °C for 20

minutes in the dark and under ambient atmosphere.[8] Films were then cooled to room temperature by turning off the hot plate.

Doping Procedure: A 50 mM solution of $FeCl_3 \cdot 6\ H_2O$ (Alfa Aesar, 97%) in acetonitrile (99.8%, Sigma Aldrich) was prepared and then serially diluted with acetonitrile to the desired doping concentration. To dope the films, 50 μL of the $FeCl_3$ dopant solution was dropped onto a ~ 1 cm^2 film. The dopant solution soaked the film for three minutes and was coved by a glass Petrie dish to mitigate solvent evaporation. After three minutes, the dopant solution was pipetted off, and the film was then rinsed with excess acetonitrile (> 100 μL). Excess solvent was removed via vacuum drying for three minutes at ~ 1 Torr and room temperature.

Thermoelectric Measurements: Thermoelectric measurements were performed immediately after doping to mitigate the effects of ambient atmosphere and time on the resulting thermoelectric properties.[9] Electrical conductivity and Seebeck coefficient measurements were performed on a custom set up, using temperature-controlled Peltier units and tungsten tipped micromanipulators. Electrical conductivity measurements were performed using the Van der Pauw technique on top of a single Peltier unit. The micromanipulators made electrical contact by piercing through the PBTTT films to the bottom gold contact pads, spaced *ca.* 5 mm from one another. For temperature dependent measurements, the electrical conductivity was measured from *ca.* 15 °C to 35 °C in steps of 2.5 °C to mitigate thermal degradation and calculate the activation energy in the vicinity of the nominal $S(\sigma)$ values reported in the main text. The Seebeck coefficient was measured by suspending the film between two Peltier stages and applying a series of temperature gradients (up to ΔT = 5 °C, centered about the central temperature) across the film. The resulting thermoelectric voltage was measured across two gold contact pads, and the temperature of each contact pads was measured using K-type thermocouples. The Seebeck coefficient was calculated from the slope of the thermoelectric voltage as a function of the applied ΔT. Over this doping ranges and temperature range, *ca.* 15 °C to 35 °C, Seebeck coefficients were temperature independent. All measurements were captured using a Keithley 2700 DMM and a 7708 Mux card via a LabView interface. Film thicknesses were measured using a ProFilm optical profilometry by measure the step height across a small scratch in the film after the doping and thermoelectric measurements steps.

UV-Vis-NIR Measurements: An Avantes fiber optic spectrometer (Avaspec-ULS2048CL-EVO-RS) with a deuterium-tungsten, halogen light source (Mikropack DH-2000) was used in transmission mode to collect ultraviolet-visible-near infrared (UV-vis-NIR) optical measurements on glass substrates immediately after doping. Film thicknesses were measured using a ProFilm optical profilometry by measure the step height across a small scratch in the film. Thickness values were needed to calculate the attenuation coefficient values in **Figure 1b**.

GIWAXS Measurements: PBTTT films were deposited on undoped Si wafers with a native oxide layer, akin to previously described. Films were measured at Soft Matter Interfaces Beamline (SMI, Beamline 12-ID) at the National Synchrotron Light Source II at 12 keV. X-ray scattering patterns were recorded on a Pilatus 900 K−W detector, consisting of 0.172 mm square pixels, mounted at a fixed distance of 0.279 m from the sample position. To cover the range of scattering angles desired, the vertically oriented elongated detector was moved horizontally on a fixed arc and images were later visualized in Xi-CAM software[10] and stitched using custom code. The sample and detector were enclosed in a vacuum chamber to suppress air scatter. Scattering patterns were measured as a function of angle of incidence, with data shown acquired with an angle of incidence near the critical angle that maximized scattering intensity from the sample.

Spectroscopic Ellipsometry (SE) Measurements: SE measurements were performed on a Woollam Alpha-SE ellipsometer at an incident angle of 70° and analyzed using CompleteEASE software. Scotch tape was adhered to the backside of the glass substrate to mitigate multiple reflections between the substrate, film,

and metal sample stage. A Cauchy model for the bare glass substrate with scotch tape (no PBTTT film) was developed and then used for subsequent modeling.

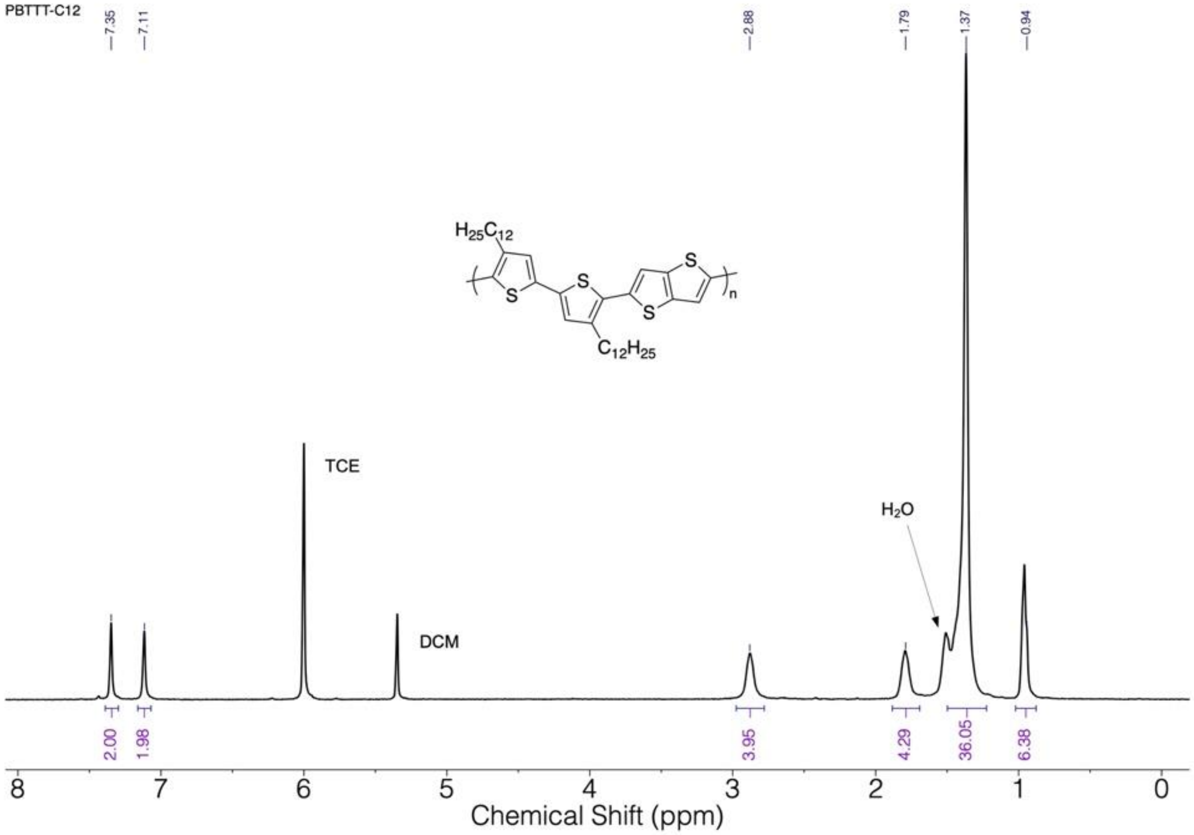

Figure S1: PBTTT ^{1}H-NMR spectrum in TCE-D_2 at 100 °C.

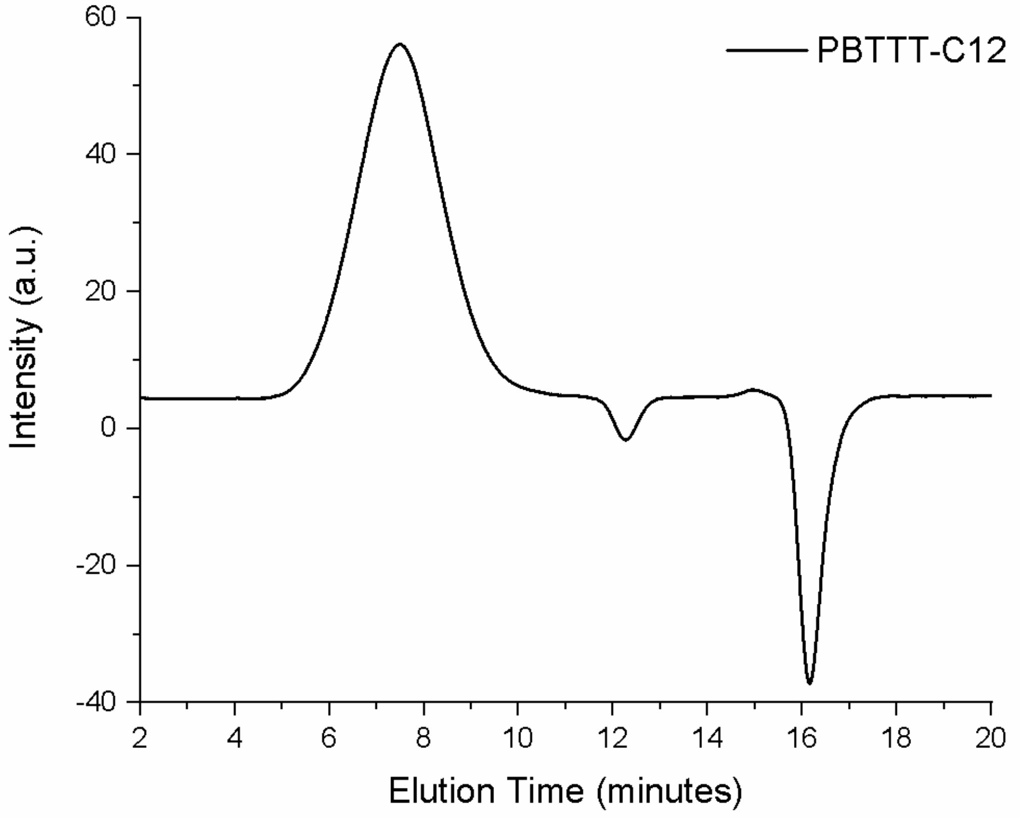

Figure S2: PBTTT GPC chromatograph in TCB at 140 °C.

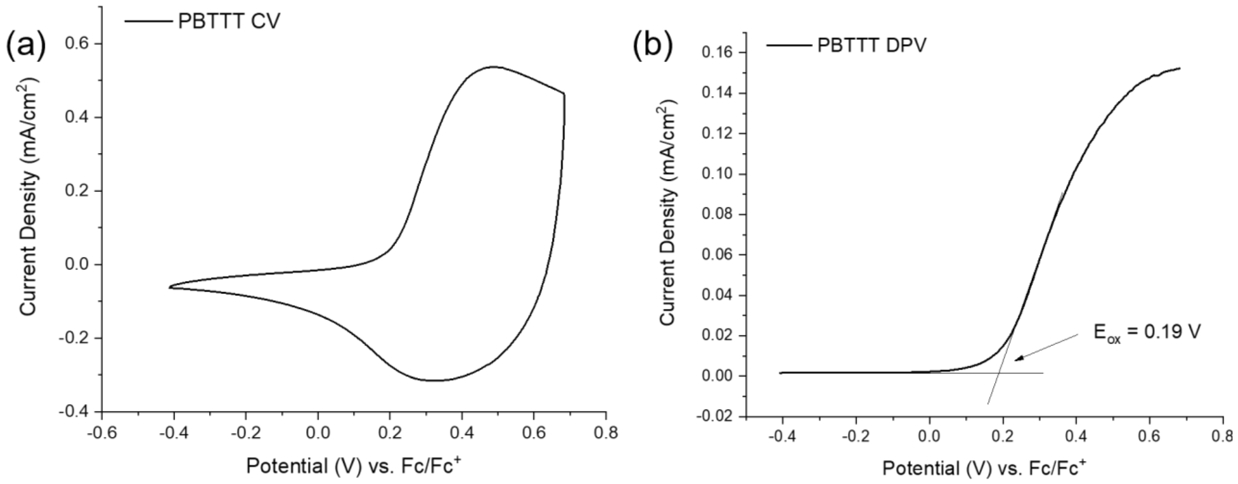

Figure S3: Electrochemistry of a PBTTT film on a glassy carbon button electrode in $TBAPF_6$/PC. (a) CV. (b) DPV.

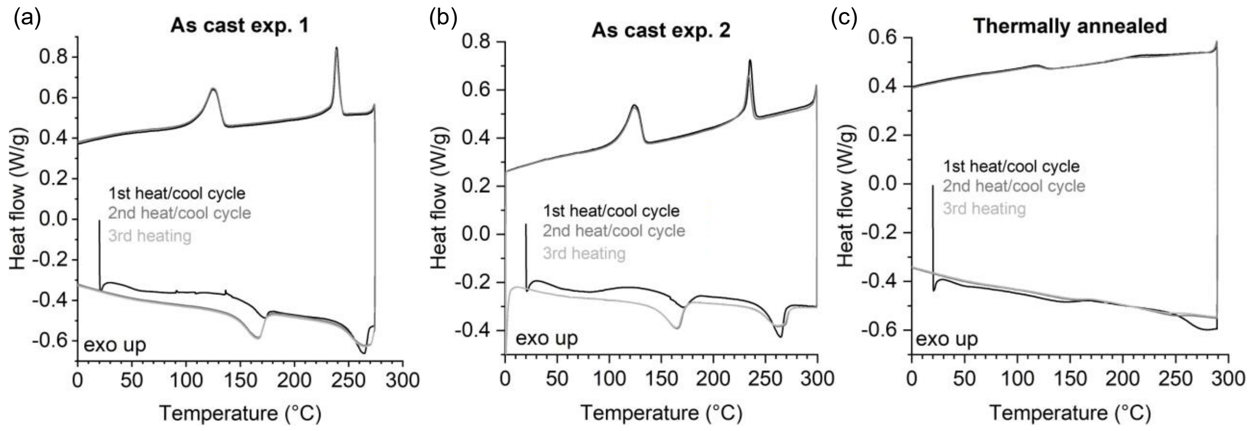

Figure S4: DSC measurements of PBTTT. (a) as cast and cycled from -50 C to 275 °C. (b) re-solvated and remeasured cycling from 0 to 300 °C. (c) Thermally annealed akin to the wire-bar films and cycled -50 to 290 °C.

Note S2: UV-Vis-NIR Peak Deconvolution for Carrier Concentration Calculations

UV-Vis-NIR measurements on PBTTT thin films were measured in transmission mode. The percentage of transmission ($\%T$) is defined as the ratio of the attenuation of light intensity due to the film $\left(\frac{I(\lambda)}{I_0(\lambda)} \times 100\%\right)$ and was converted to absorbance (A), using Beer-Lambert law, $A = 2 - \log_{10}(\%T)$. The absorbance of a film is a function of the concentration (c), extinction coefficient (ϵ), and path length (l) ($A = c\,\epsilon\,l$), and the path length is equal to the film thickness in this experimental set up.

Figure 2b plots the measured absorbance normalized by the measured film thickness $\left(\frac{A}{l}\right)$ in units of cm^{-1} for each doping level, and this is defined as the attenuation coefficient. We calculate that value of the attenuation coefficient for pristine PBTTT, 0.7 x 10^5 cm^{-1}, is comparable to other pristine conjugated polymers when measured using this method.[11-13] Note that these attenuation coefficients were performed using transmission mode UV-Vis-NIR with log-based-10 procedures. These methods and formalisms are not necessarily consistent with those used with ellipsometry. Oftentimes with ellipsometry, the reflectance, absorption, and transmission contributions are accounted (via the changes in the reflected polarized light phase and intensity) and natural logarithms (log-based-e) are used.

PBTTT doped with $FeCl_3$ has $FeCl_4^-$ counteranions.[8] If each polaronic charge carrier has a single counteranion, and these counteranions have a fixed charge and molecular stoichiometry, then the $FeCl_4^-$ absorbances at *ca.* 3.4 and 3.9 eV can be used to quantify the concentration of $FeCl_4^-$ and polaronic charge with respect to the concentration of BTTT monomers. An extinction coefficient of 50,000 L mol^{-1} cm^{-1} for PBTTT was used[12] in conjunction with the pristine PBTTT film thickness and absorbance to define the concentration of BTTT monomers in the films (see Note S1 of Ref.[13]). Extinction coefficients of 8,100 and 7,400 L mol^{-1} cm^{-1} for the 3.4 and 3.9 eV peaks of $FeCl_4^-$ were used, respectively (see Table S4 of Ref.[8]).[8] These $FeCl_4^-$ extinction coefficients were used in conjunction with the deconvolved attenuation coefficient peak heights to calculate the $FeCl_4^-$ concentrations. Peak deconvolutions were necessary because the peak shape and baseline shifted as a function of doping level.[8, 14] At each doping level, the peak height was calculated using a mixed Gaussian-Lorentzian peak deconvolution using Origin 2021 software. **Figure S5,6** shows representative deconvolutions.

The calculated molar ratios of $FeCl_4^-$ with respect to PBTTT ranged from 0.11 to 0.40, and these molar ratios are comparable to previous reports.[8, 14] Carrier ratios were calculated by assuming that each PBTTT repeat unit can support two charge carriers (*i.e.* two carriers repeat unit).[15] Carrier densities were calculated using a repeat unit molecular weight of 694.8 g mol^{-1} and a mass density of 1.1 g cm^{-1}.[12] By using two $FeCl_4^-$ peaks for deconvolution, uncertainty error bars are provided and represent the standard deviation of the two deconvoluted peaks. This molar ratio uncertainty was propagated to carrier ratios and carrier densities in the SLoT analysis.

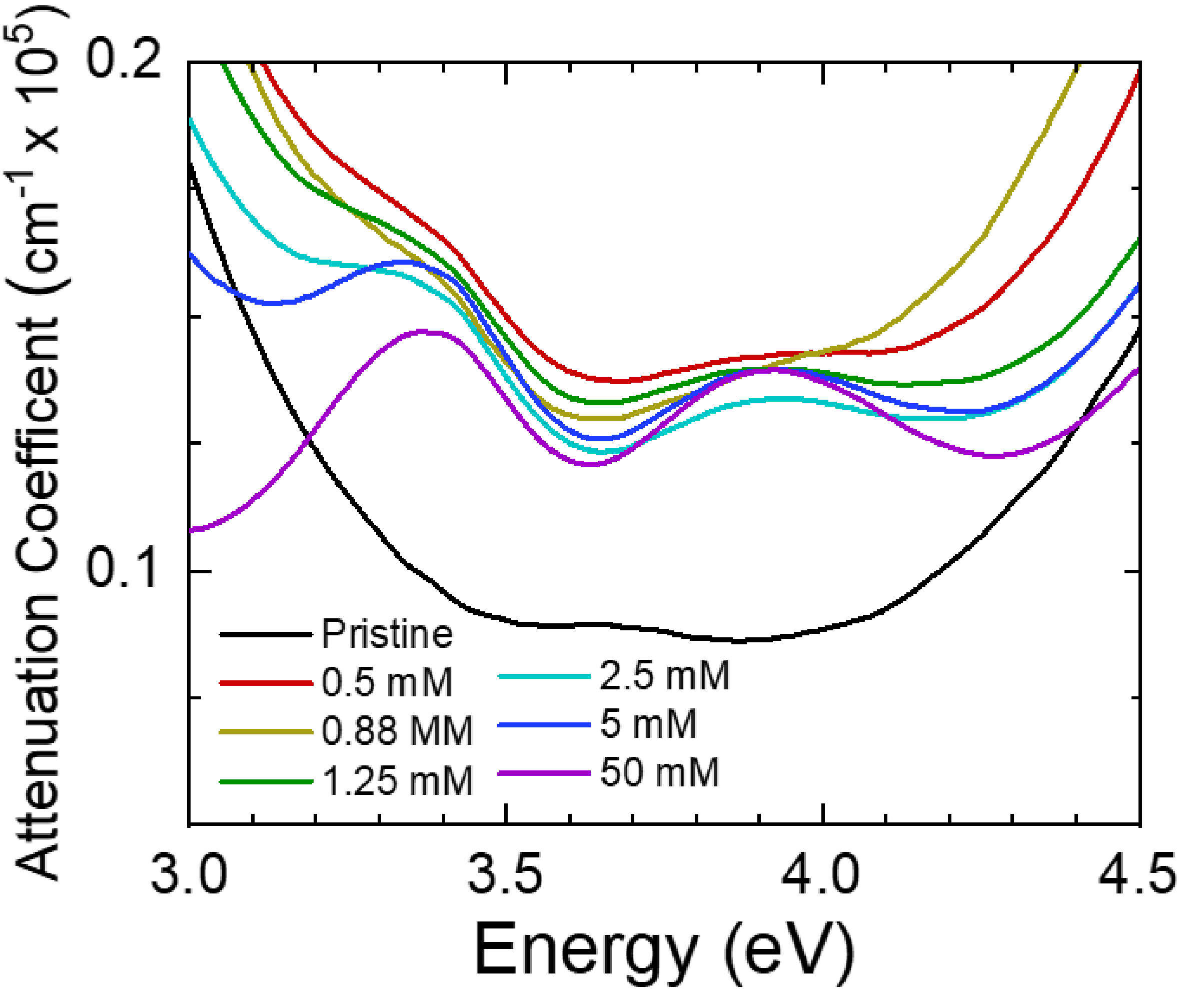

Figure S5: UV-Vis-NIR spectra used for $FeCl_4^-$ peak deconvolution.

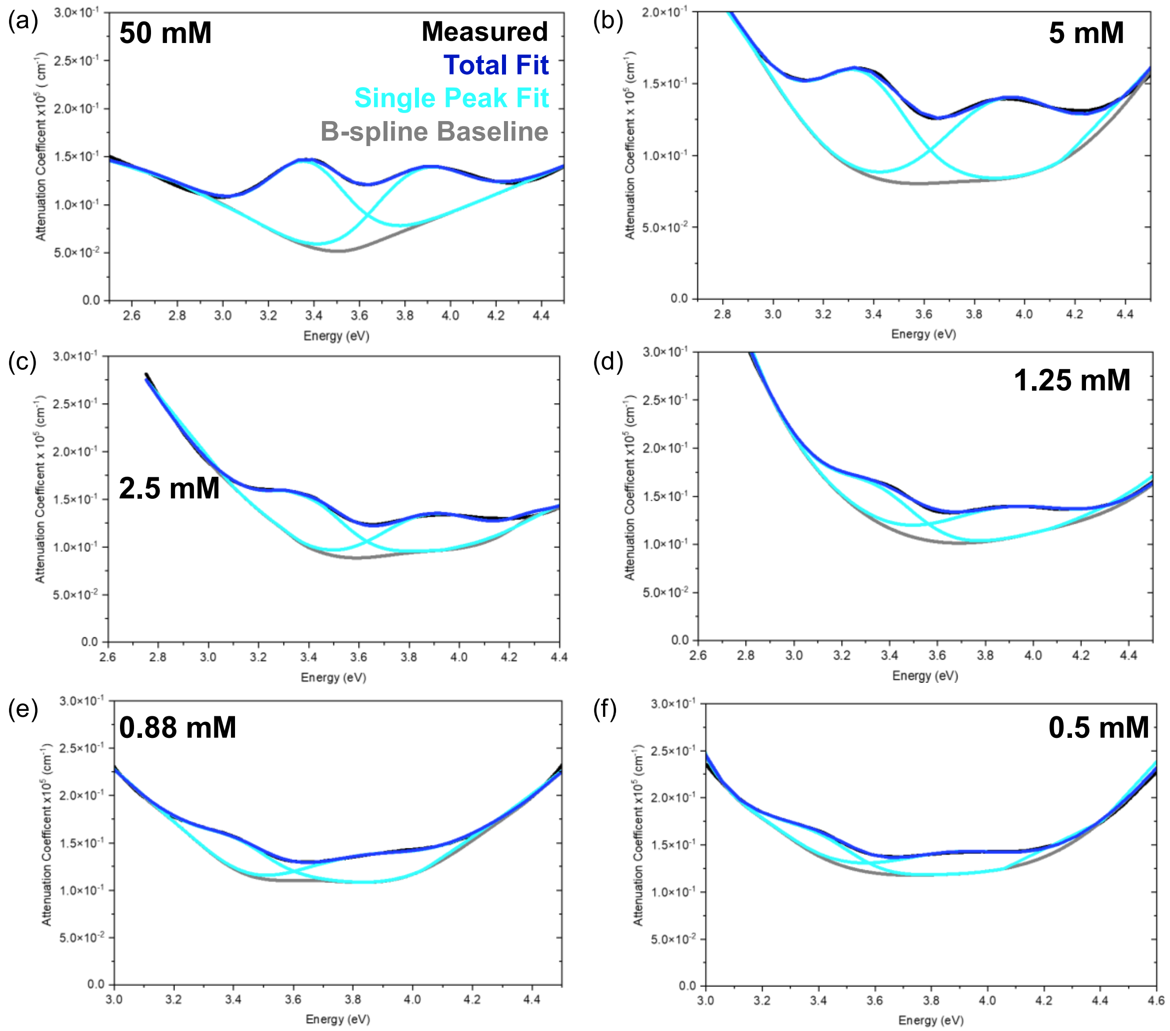

Figure S6: Deconvolutions used to calculate the attenuation coefficient peak heights and then the FeCl4- concentrations. (a) 50 mM (b) 5 mM (c) 2.5 mM (d) 1.25 mM (e) 0.88 mM (f) 0.5 mM.

Note S3: SLoT modeling notes

Figure S7 shows the XPS measurements and deconvolutions for PBTTT doped with $FeCl_3$ at varying molarities. All deconvolutions show a low χ^2 value and Abbe criterion, indicating that these deconvolutions are statistically significant and can be representative of the measured data. The pristine spectra was first deconvoluted, with a single sulfur 2p doublet, using a fixed S $2p_{3/2}$: S $2p_{1/2}$ area ratio of ~0.511 and a peak binding energy separation of ~1.2 eV, consistent with previous studies and standards.[16-18] The 0.88 mM doped film has a comparable S 2p signal to the pristine film, so a second S 2p doublet could not be prudently incorporated. Therefore the 0.88 mM film does not have an extent of oxidation that can be reasonably quantified and deconvoluted using XPS; this is in stark contrast to all thermoelectric and optical measurements which clearly show evidence of oxidation.

At 2.5 mM and 50 mM, XPS measurements notably and asymmetrically shift towards higher binding energies, indicative of oxidative doping. Because of binding energy and area ratio constraints, a second set of doublet peaks are needed to capture the signal at higher binding energies. The area of these oxidized doublets with respect to the total sulfur area is used to quantify the extent of sulfur oxidation. At 2.5 mM, the oxidized sulfur : total sulfur area ratio is 0.09 or ~1 out of 10 sulfurs are oxidized. At 50 mM, the oxidized sulfur: total sulfur area is 0.24, or ~1 out of 4 sulfurs are oxidized. With 4 sulfurs per monomer unit, these XPS results are commensurate with a dopant : monomer ratio of 0.36 and 0.96, respectively, and a carrier ratio of 0.18 and 0.48 (assuming 2 sites per monomer), respectively. For reference, the UV-Vis-NIR peak deconvolutions yielded carrier ratios of 0.22 ± 0.06 and 0.40 ± 0.04, which is consistent within error of the XPS measurements. Additionally, we note that XPS survey measurements and elemental spectra for iron and chlorine substantiate the use UV-Vis for $FeCl_x$ concentration quantification (**Figure S8, Table S1**). Notably, PBTTT having a maximum extent of doping of 1 out of 4 sulfurs oxidized is still low compared to P3HT (~1 out of 3) and PE_2/PEDOT (~ 1 out of 2),[16, 19] and an order of magnitude lower than that modeled used spectroscopic ellipsometry or AC Hall effects.[14]

We also note that the intensity-weighted average binding energy (E_W) shifts are reasonable and consistent with the η values calculated using the SLoT model. If the reference vacuum energy level is constant, and the shift in core energies is equal to the shift in valence energies (as shown in Ref.[18]), then the shift in the weighted binding energies is commensurate to the shift in E_F. Mathematically, this can be expressed as, $E_w = \frac{\sum_{E_1}^{E_2} \text{Intensity x Binding Energy}}{\sum_{E_1}^{E_2} \text{Intensity}}$, where the bounds of E_2 and E_1 are the binding energy bounds (ca. 161 and 168 eV for S-2p in this study). Similarly, if the transport edge is constant and the transport properties are well modeled using a linear transport function, then the shift in η is commensurate to the shift in E_F . Explicitly, at 0.88 mM, the E_w is 164.36 eV with an η of 1.5. At 50 mM, the E_w is 164.67 eV and η of 14. Therefore, as the extent of doping increases from 0.88 mM to 50 mM, the ΔE_w= 0.31 eV and the $\Delta\eta$ = 12.5 = 0.33 eV at 300 K. Because $\Delta E_w \sim \Delta\eta$, we assert that the asserted transport function (and its localized prefactor, see Eq. 1-3) are reasonable and self-consistent with the spectroscopic observations.

Figure S9a shows that σ_0 does not have a statically significant linear slope (p-score > 0.05) and therefore does not significantly vary as a function of doping level. This invariance is in stark contrast to σ_{E_0} which shows an exponential dependence on doping level and reinforces the need to quantify W_H. Therefore, we assert that σ_0 can be used to describe the entire PBTTT-$FeCl_3$ series studied herein. A single σ_0 parameter is useful because it quantifies the propensity for high electrical conductivities and mobilities for a system, independent of doping level, and it can be related to energy-independent contributions to charge transport (*e.g.* effective mass in a parabolic band approximation).[20]

Because σ_0 is constant, we examine the distribution of σ_0 values calculated for each film. Each films' σ_0 is represented by a single small black box in **Figure S9b.** The large grey box represents the average value ± one standard deviation, and whiskers represent two standard deviations. The star represents the median value, and a simple Gaussian curve is overlayed. Asserting that σ_0 = 25 S cm^{-1} is consistent with the distribution shown in **Figure S9b**, fits the experimental $S(\sigma)$ curve (with the asserted $W_{\mathrm{H}}(c)$ and $\eta(c)$ curves), and is consistent with our previous predictions.[16]

Figure S10 shows our previous models and predictions regarding charge transport in chemically doped PBTTT. The SLoT model parameters used for the predicted curves are found in Ref.[16], the PBTTTC14 data is found in Ref.[15], and the PBTTTC16 data is found in Ref.[21]. From these literature reports, we previously predicted the σ_0 and SLoT curve from solely $S(\sigma)$ data. Here we fully evaluated the SLoT model and found consistency with our previous model predictions.

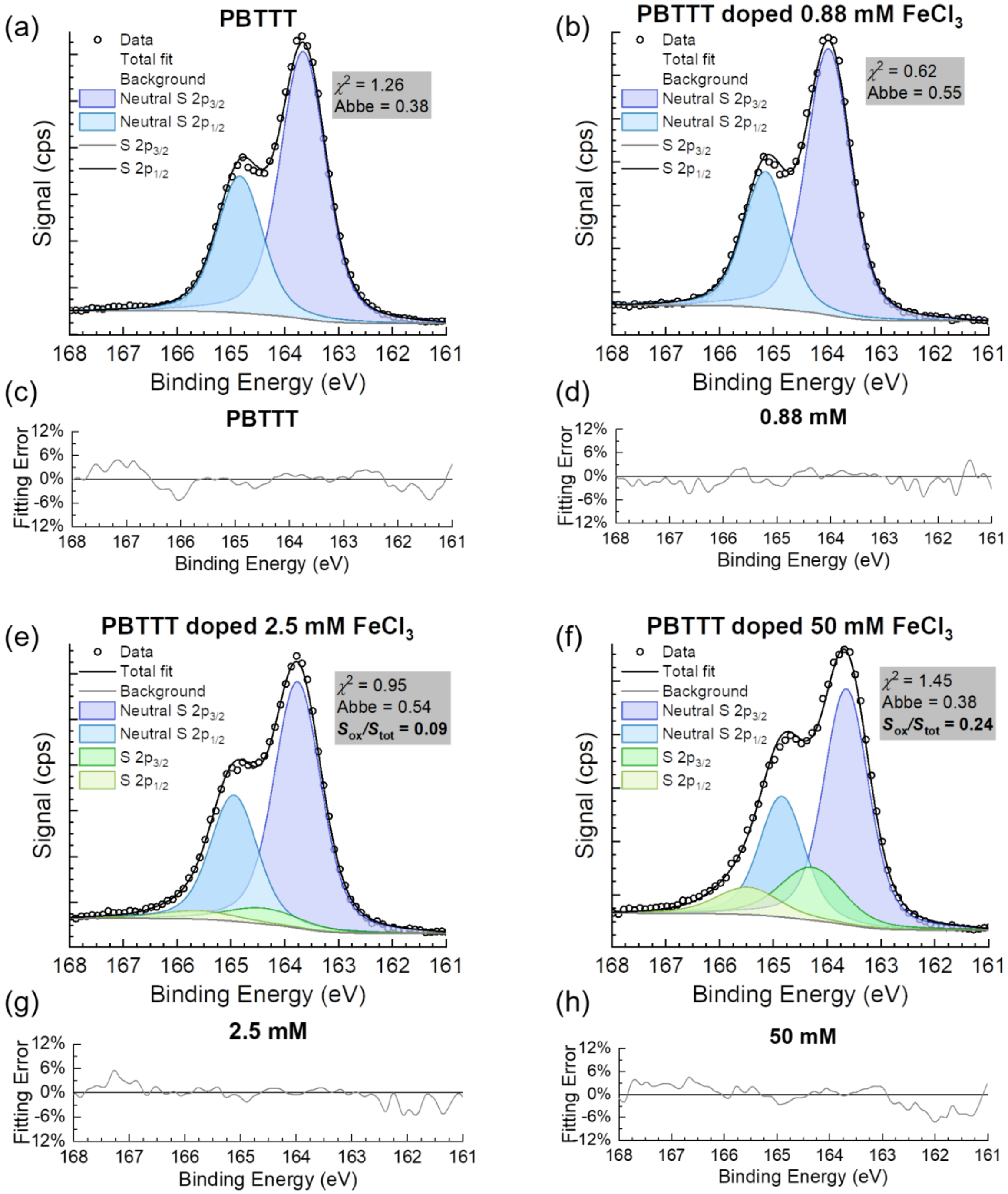

Figure S7: X-ray photo electron spectroscopy measurements and deconvolutions for representative doping levels. (a,b,e,f) measured and deconvoluted spectrograms. (c,d,g,h) calculated fitting errors as calculated from residuals/ envelope x 100%

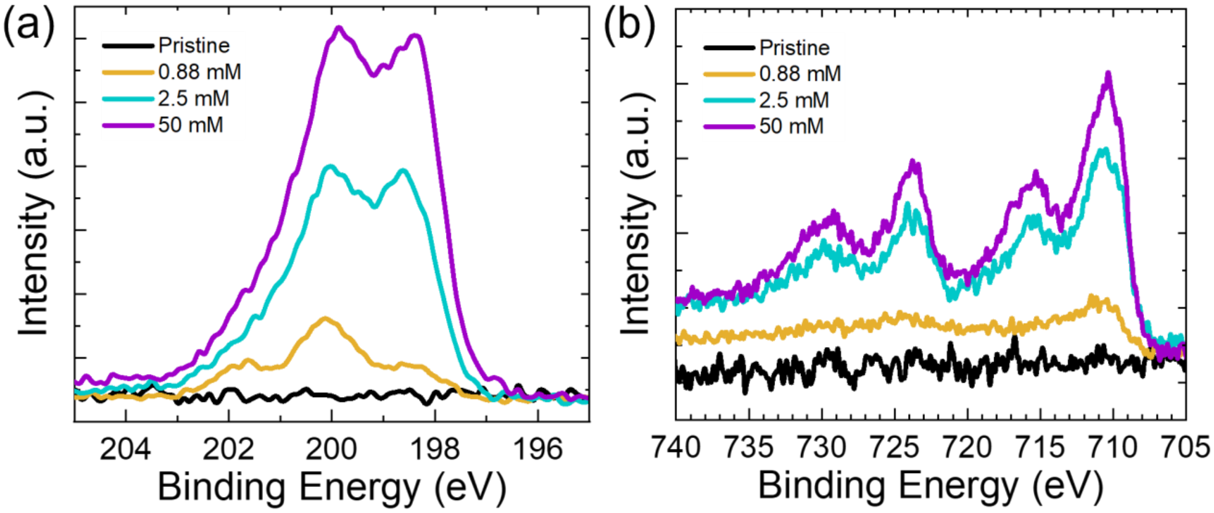

Figure S8: X-ray photo electron spectroscopy measurements for (a) chlorine and (b) iron. Vertical axes are offset to facilitate comparisons between spectra.

Table S1: XPS Survey Atomic Abundances

System	C-1s	S-2p	O-1s	Cl-2p	Fe-2p
Pristine	88	9.4	3.0	-	-
0.88 mM	82	8.0	5.1	3.5	0.98
2.5 mM	85	9.0	3.3	2.1	0.96
50 mM	83	8.9	3.0	3.6	1.1

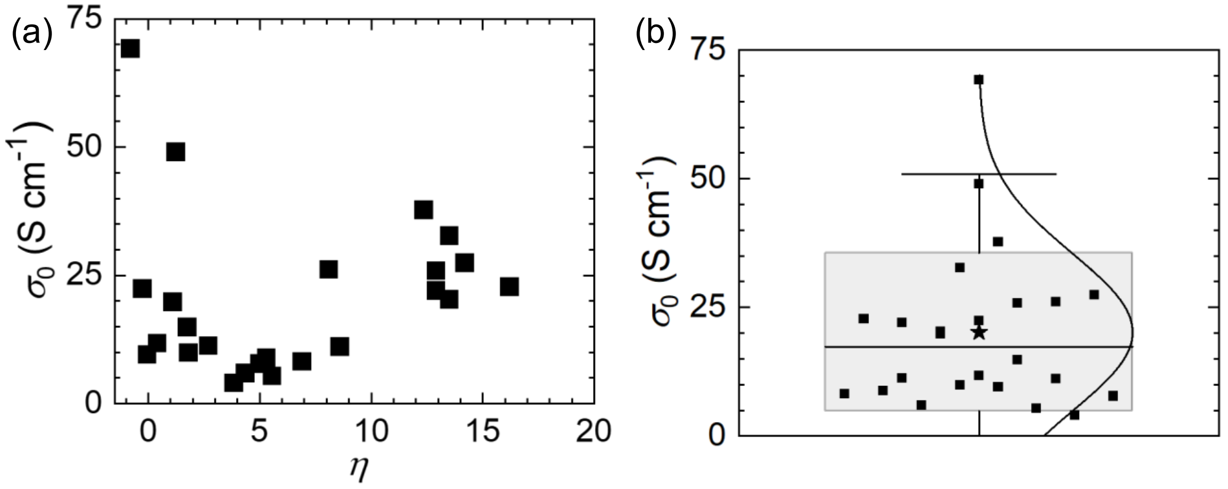

Figure S9: σ_0 evaluations. (a) $\sigma_0(\eta)$. (b) Distribution of σ_0.

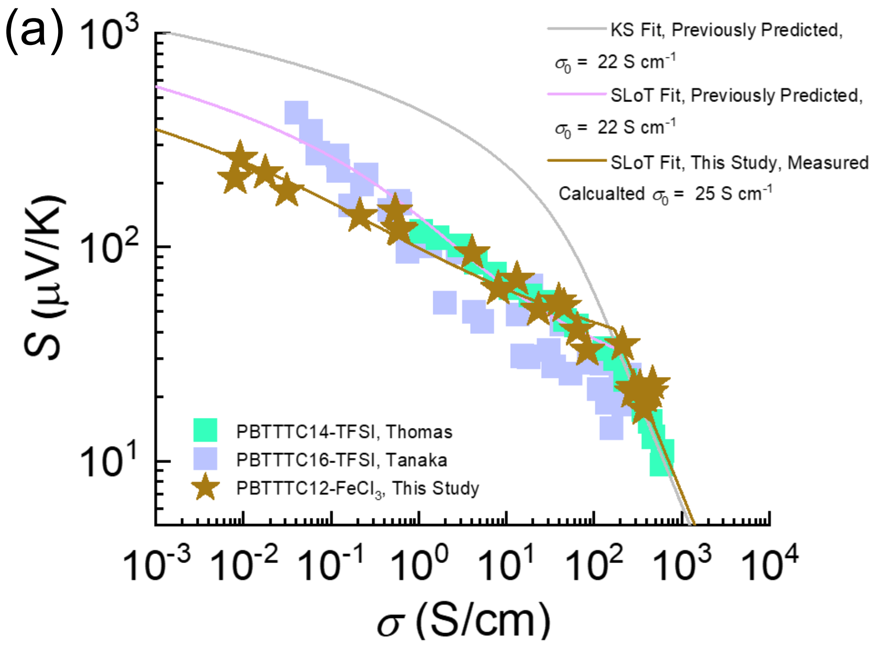

Figure S10: Comparison of PBTTT SLoT predictions with SLoT calculations.

Note S4: GIWAXS

Figure S11 shows a representative peak assignment from a radially integrated GIWAXS pattern. Peaks were deconvoluted using Origin 2021 software, with an asymmetric least squares background fit and a Lorentzian-Gaussian cross peak shape.

Two common tools to analyze the size and quality of a crystalline domain in conjugated polymers are the coherence length (L_c) and paracrystallinity (g).[7, 22-24]

Oftentimes, the coherence length can be calculated using the Scherrer equation $\left(L_c = \frac{2\pi K}{\Delta q}\right)$, where K is a shape factor (near 0.9), and Δq is the full width at half-max (FWHM) in q-space for a diffraction peak.[22] This Scherrer equation is best used for inorganic crystalline materials that do not have disorder, where the peak width is only affected by the crystal size. In these ideal crystalline systems, the FWHM is nearly constant for all higher order reflections, *e.g.* (200), (300), *etc.* In contrast, semiconducting polymers have cumulative disorder (predominantly paracrystalline disorder). With cumulative disorder the polymer chains have an increasing propensity to change spatial coordinate and lose periodicity. Therefore, as the reflection order increases, the FWHM increases, as shown in **Figure S12a**.

To isolate the effects of paracrystalline disorder on the (h00) lamellar peak, the FWHM is plotted as a function of h^2, and L_c is the calculated y-intercept. At the y-intercept, the effects of disorder are minimized (mathematically, this can be thought as a zero-order reflection). **Figure S12b** shows that as h^2 increases, the FWHM increases linearly. Note that a (400) peak was not deconvoluted for the 50 mM PBTTT film, as the (400) peak was not isolatable from (003) peak.

Table S2 shows the L_c values calculated using simple Scherrer equation with the (100) peak and the L_c values calculated using the linear intercept method. Both methods show that doping decreases L_c in the (h00) direction, consistent with previous reports.[25] Furthermore, we note that when performing linear regressions, it is important to report the range of the confidence interval (or some error approximation) and the statistical significance (p-score). Here, we find that the p-score for the intercept is greater than the confidence level (0.05) in most films. Therefore, there is a large degree of uncertainty around L_c values reported using the linear intercept, *i.e.* statistically insignificant. This is also reflected in the large range of permissible values within the 95% confidence intervals (CI). This statistical analysis weakens the argument that doping affects L_c, as calculated using the intercept method.

Regarding the (110) peak, for the $\pi - \pi$ stacks, the paracrystallinity g values is oftentimes reported because higher order (h10) peaks are not oftentimes observable, making the intercept method unusable. Additionally, we note that we attribute this peak to the (110) Miller index, and not (010) because PBTTT has a triclinic unit cell.[26] It can be shown for highly disordered systems that $g = \frac{1}{2\pi}\sqrt{\Delta q \cdot d}$, where d is the real spacing of the (110) peak; note that this equation assumes that the effects from lattice-parameter fluctuation can be neglected.[23] For context, g values range for highly crystalline small organic molecules (pentacene, $g\sim$ 5%) well-ordered conjugated polymers (PBTTT/P3HT, $g\sim$ 5-15%), disordered conjugated polymers (PCDTBT , $g\sim$ 15-25%) and amorphous (PMMA, , $g >$ 25%)[7] We find that the g value of this PBTTT is quite small, *ca.* 5.5 %, which indicates high ordering within the crystalline domains.

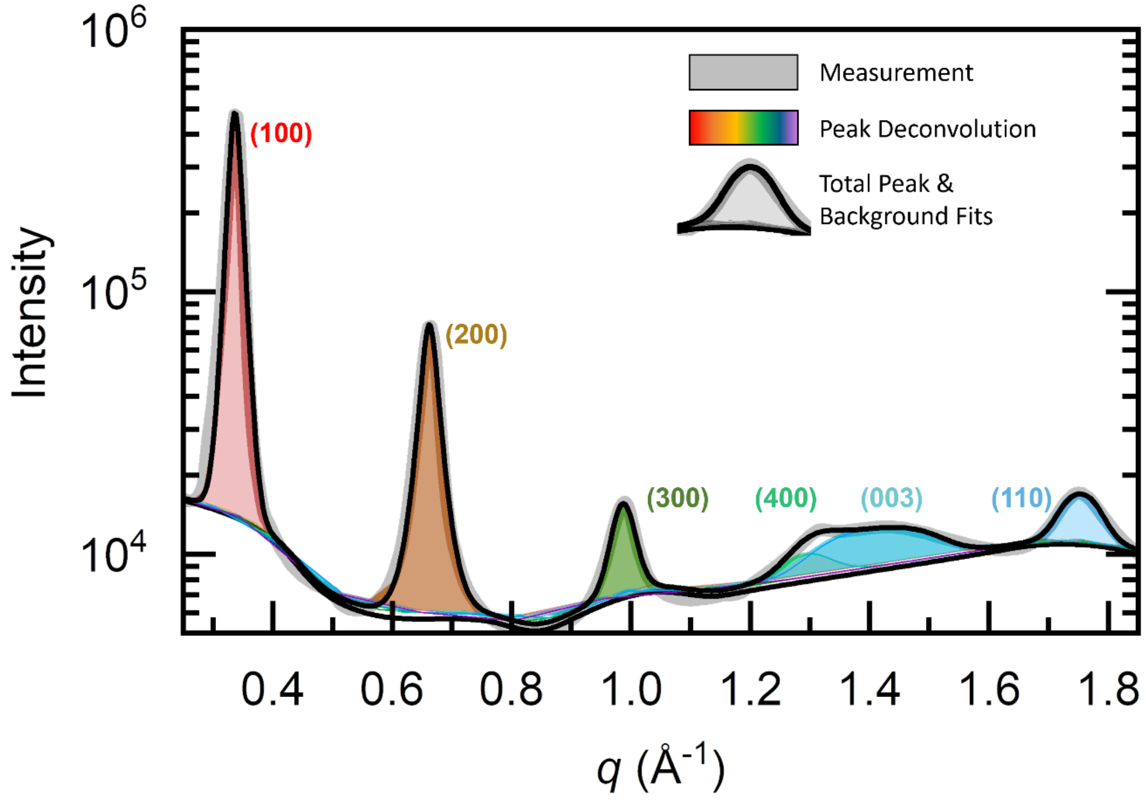

Figure S11: Representative GIWAXS deconvolution. Raw data (grey curves) was fitted by a series of Gaussian-Lorentzian curves (colored and shaded areas). Black curves represent the baseline and total peak fits.

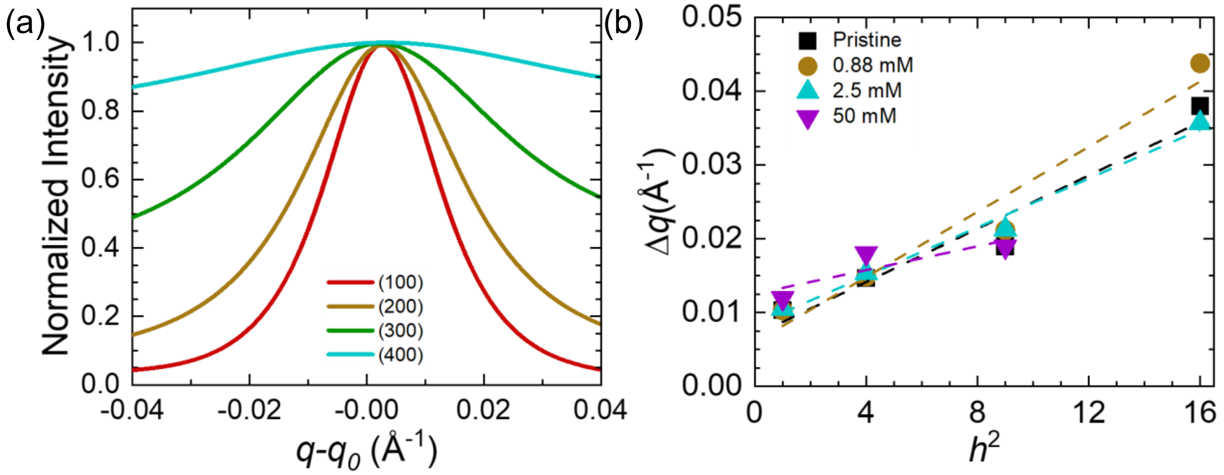

Figure S12: GIWAXS Analysis. (a) Representative plot showing that the peak broadens with respect to the peak reflection maximum location (q_0). (b) Δq as a function of the (h00) reflection.

Table S2: Coherence Length tabulations

System	L_c (nm), (100)	L_c (nm), Intercept	95% CI	*p*-score	L_c (nm)(110)
Pristine	61	91	± 42%	0.14	20.6
0.88 mM	61	105	± 56%	0.21	18.6
2.5 mM	60	75	± 15%	0.023	17.6
50 mM	53	52	± 22%	0.14	17.6

Note S5: Spectroscopic Ellipsometry and Attenuated Total Reflectance Infrared Absorbance Measurements

Figure S13 shows the spectroscopic ellipsometry measurements. Note that all ψ and Δ axes are the same scaling to help comparisons, but individual films ranged from ca. 60 to 250 nm which can affect the observed ψ and Δ. Measured data points are represented by hollow data points and only every fifth data point is shown for clarity. Solid lines represent B-spline model fits, calculated using an optical model. This optical model was developed using known film thicknesses (measured using optical profilometry) and known glass substrate optical properties (measured using ellipsometry). The B-spline model fits assumed Kramers-Kronig consistency. This optical model was developed using the CompleteEASE software (J.A. Woollam).

Figure S14 shows the calculated $\tilde{n}, \tilde{k},$ and α values. $\tilde{n}$ is the real part of the index of refraction, $\tilde{k}$ is the imaginary part of the index of refraction, and α is the absorption coefficient. We use tilde (~) above n and k here to distinguish the index of refraction values from carrier densities and wave vectors. These values are related to ϵ_1 and ϵ_2,[27]

$$\epsilon_1 = \epsilon_0\,(\tilde{n}^2 - \tilde{k}^2) \quad \text{(S1)}$$

$$\epsilon_2 = \epsilon_0\,(2\tilde{n}\tilde{k}). \quad \text{(S2)}$$

The pristine $\pi - \pi^*$ transition obtaining a maximum $\tilde{n}$ value near 3 is consistent with literature reports on conjugated polymers.[28, 29] Additionally, pristine PBTTT obtaining a maximum $\tilde{k}$ value near 1.5 is consistent with previous reports.[28] Notably, it has been shown that the complex index of refraction increases significantly with increasing molecular weight in conjugated polymers.[28]

The absorption coefficient, α is calculated as,[28, 30]

$$\alpha = \frac{4\pi\tilde{k}}{\lambda} \quad \text{(S3)}$$

where λ is the wavelength. Absorption coefficients ought to be asserted only when contributions from reflection, transmission, and absorbance are measurable and/or calculable. For example, transmission UV-Vis-NIR measurements alone may not be able to assert α values because the reflectance is not measured, and the commonly reported absorbance assumes that all light is either absorbed or transmitted. In contrast, spectroscopic ellipsometry uses the changes in reflected light intensity and phase of *s*- and *p*- polarized light in a system of equations.[27] These nuanced differences in measurement is why in **Figure 2** we asserted attenuation coefficients while in **Figure S14** we asserted absorption coefficients (α). Furthermore, the absorption coefficient is calculated from measured intensities using natural logarithms (*i.e.* $I(l) = I_0 \exp(-\alpha l)$). These subtle differences undermine the importance of clearly reporting optical measurement conditions and assumptions and to judiciously make fair comparisons between comparably obtained values.

In a series of electropolymerized dioxythiophene derivatives, measured using both reflectance and transmission measurements, α for the pristine $\pi - \pi^*$ peak near 2-2.2 eV ranges from 1.5 - 2 x 10^5 cm^{-1}.[31, 32] In a series of thieno[3,2-b]thiophene-diketopyrrolopyrrole (DPP-TT-T) polymers measured using spectroscopic ellipsometry, α for the pristine $\pi - \pi^*$ peak near 1.5-2 eV ranges from 2.2 - 3 x 10^5 cm^{-1}.[28] Notably, in Ref.[28], the authors found that polymers with long, colinear, planar monomers have enhanced α values. Lastly, we note that for this PBTTT system, the amount of paracrystalline disorder is exceptionally low (~ 5.5 %),[7] which indicates comparatively high levels of ordering in the $\pi - \pi$ stacks, and this could

be responsible for the large α values. Considering these literature comparisons and the additional characterizations performed herein, we believe that the calculated $\tilde{n}, \tilde{k},$ and α values are reasonable.

Figure S15 shows the ϵ_2 deconvolutions for the 50 mM $FeCl_3$ doped PBTTT films. The deconvolution with the Drude contribution has a mean squared error (MSE) of 3.4. The deconvolution without the Drude contribution, has a MSE of 13.9. This suggests that the Drude contribution improves the model fitting. Qualitatively, it is seen that the model fitting without the Drude contribution has steeper slopes and larger amplitudes for the polaronic and $\pi - \pi^*$ transitions. A Drude-like asymptotic tail reduces these effects.

Figure S16 shows the attenuation coefficient and approximated imaginary part of the refractive index. Note that these measurements were performed using attenuated total reflectance- infrared spectroscopy (ATR-FTIR), with no complimentary transmission or reflectance measurements. Therefore, we caution precise quantitative comparisons in these IR spectra, but encourage semi-quantitative and qualitative analysis. FTIR-ATR measurements were performed on a Thermo Fisher Scientific Nicolet iS5 FTIR with an iD7 Diamond ATR attachment. Before each measurement, background scans were collected on an empty ATR stage and software subtracted from the measured spectrum. PBTTT films were measured on glass slides by placing the film side "face down" onto the ATR crystal and mechanical pressure was applied to ensure contact between the film and ATR crystal. Films were sufficiently thin so that the ATR measurement measured the through the film thickness.

In **Figure 16a**, the attenuation coefficients are reasonable and within a factor of 2 of the absorption coefficient (α) reported for XDOT derivatives using both transmission and reflectance techniques.[31, 32] Furthermore, these attenuation coefficients at 0.6 eV are approximately equal to those at 1.4 eV measured using spectroscopic ellipsometry. The pristine spectra show CH_x absorbances (near 3000 cm^{-1}) associated with the hydrocarbon sidechains in PBTTT. These CH_x peaks are observable across most doping levels, but a Drude-like and/or (bi)polaronic band emerges in the mid-infrared (MIR) that obfuscates these peaks at higher doping levels. As polaronic charge carriers become more delocalized and mobile, a broad MIR peak (between 0.2 and 1 eV) emerges and shifts to lower energies (associated with intrachain transport), and the peak at 0.17 eV (associated with interchain transport) increases in amplitude with respect to the interchain peak.[33, 34] Initially, the intrachain MIR peak redshifts and the interchain peak increases in amplitude with respect to the MIR peak (*ca.* pristine through 1.25 mM). Beyond 1.25 mM, these polaronic trends are not consistent with the experimental observations. The attenuation coefficients continue to broaden into linear slopes as the molarity increases from 1.25 mM to 50 mM. Notably, the transition at *ca.* 1.25 mM is at the same molarity where $W_{\mathrm{H}} < k_{\mathrm{B}}T$ and the SLoT model becomes co-linear with the delocalized model (**Figure 3d**).

Also shown in **Figure 16a** are Drude model curves which show absorbance due to free carriers. Notably, these Drude model curves cannot capture the discrete oscillatory transition associated with the CH_x absorbances, and the inter/intrachain polaronic absorbances. Indeed, Lorentz-like oscillators are needed for these transitions. However, these Drude model curves can help explain the slopes and curvatures exhibited by the 1.25 mM through 50 mM curves.

The bottom most model curve, 3×10^{20} carriers cm^{-3}, was calculated using the macroscopic transport parameters. The slopes and curvatures of this model are inconsistent with the spectroscopic measurements and indicate that the macroscopic transport measurements are an underestimate of the transport properties in the most conductive domains. The topmost curve, 6×10^{21} carriers cm^{-3}, was calculated with the transport

parameters from spectroscopic ellipsometry, in the range of 1.4 to 3.2 eV. Here, we observe that the slope and curvature may be too steep to explain the MIR optical properties. This could be because of the ATR-FTIR set up and/or because the ellipsometry measurements were performed in too narrow of a spectroscopic range. Lastly, we model a middle curve, with averaged electrical conductivities and carrier densities from the other two curves. We find that this middle curve fits the 2.5 mM spectrum exceptionally well and may be a basis for explaining the other highly doped curves.

Overall, these MIR optical measurements and models confirm that there is a Drude contribution, especially at higher doping levels, and there are likely domains with higher carrier densities and electrical conductivities than observable with macroscopic transport measurements. Ultimately, performing spectroscopic ellipsometry over wider frequency ranges, more angles, and with additional polymeric systems is needed.

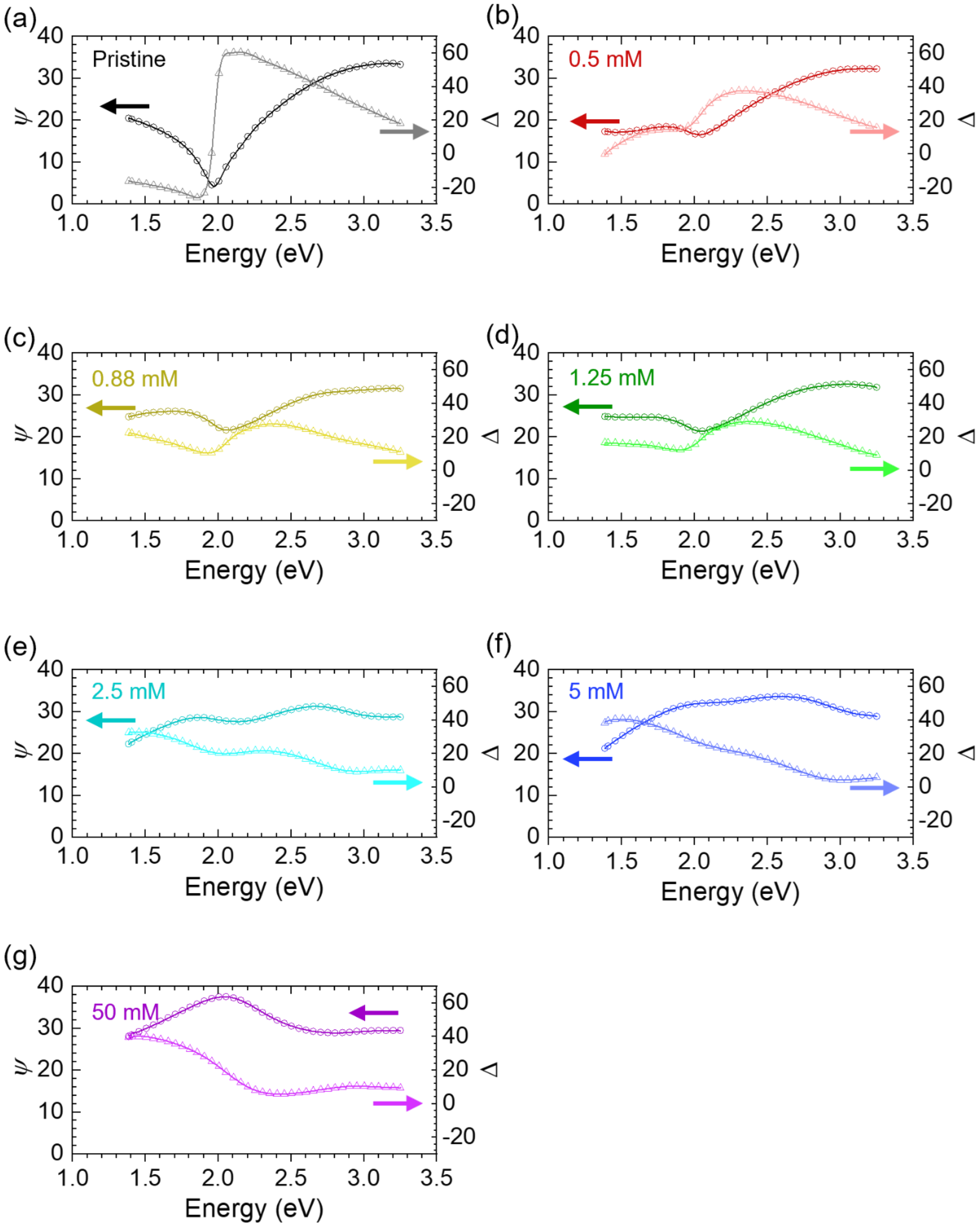

Figure S13: ψ and Δ measurements. (a) Pristine PBTTT. (b) 0.5 mM FeCl3 doped. (c) 0.88 mM. (d) 1.25 mM. (e) 2.5 mM. (f) 5 mM. (g) 50 mM.

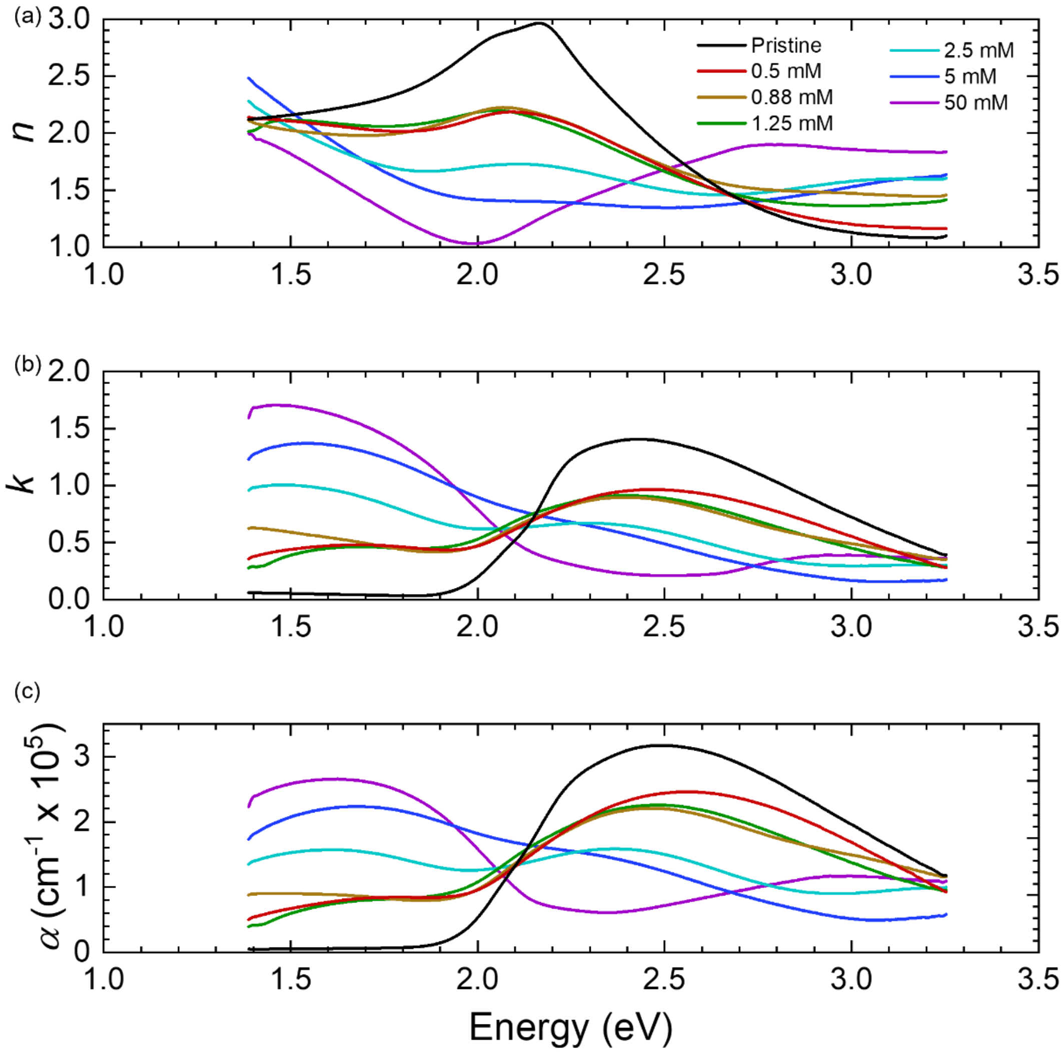

Figure S14: $\tilde{n}, \tilde{k}, \alpha$ optical constants from SE. (a) Real part of the complex refractive index. (b) Imaginary part of the complex refractive index. (c) Absorption coefficient.

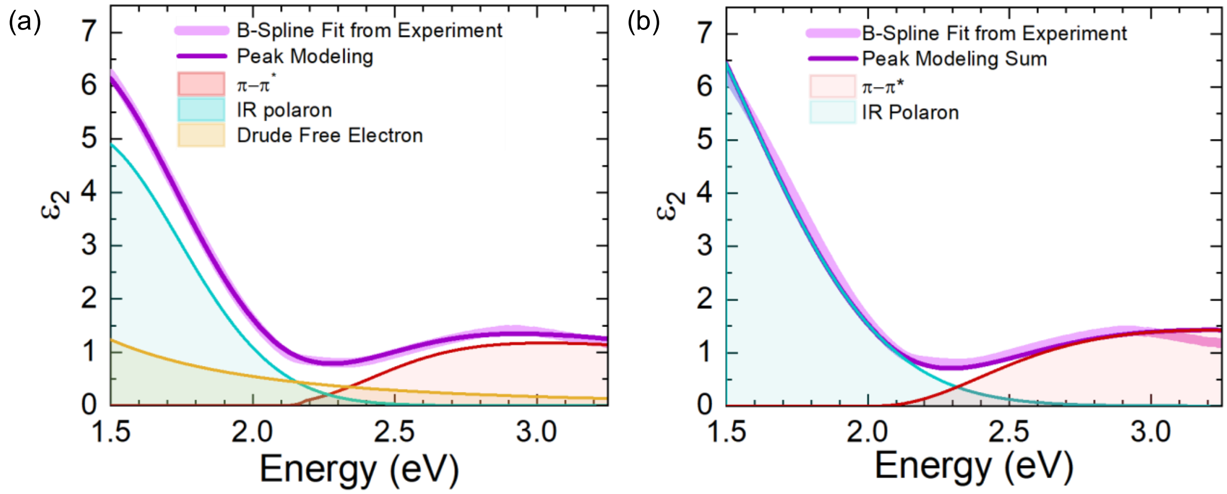

Figure S15: Fitting 50 mM PBTTT SE measurements with and without Drude free electron contributions.

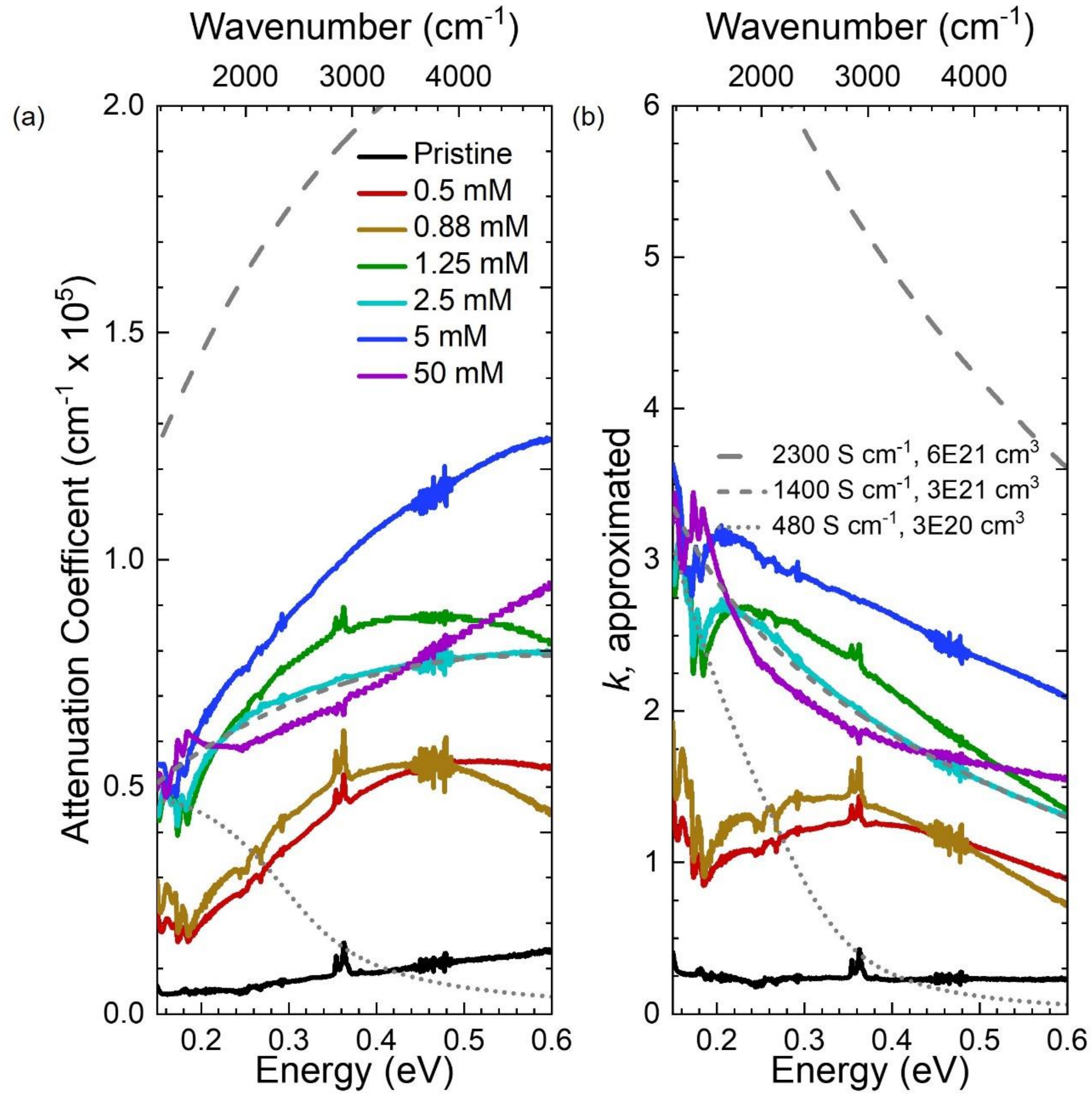

Figure S16: ATR-FTIR measurements and optical constants. (a) Approximated attenuation coefficients, calculated from the measured absorbance normalized by film thickness. (b) Approximated imaginary part of the refractive index. Note that these values are approximates, as an ATR technique was employed- not transmittance complemented with reflectance nor ellipsometry.

References

1. Zalesskiy, S. S.; Ananikov, V. P., Pd2(dba)3as a Precursor of Soluble Metal Complexes and Nanoparticles: Determination of Palladium Active Species for Catalysis and Synthesis. *Organometallics* **2012,** *31* (6), 2302-2309.
2. Vijayakumar, V.; Zaborova, E.; Biniek, L.; Zeng, H.; Herrmann, L.; Carvalho, A.; Boyron, O.; Leclerc, N.; Brinkmann, M., Effect of Alkyl Side Chain Length on Doping Kinetics, Thermopower, and Charge Transport Properties in Highly Oriented F4TCNQ-Doped PBTTT Films. *ACS Appl Mater Interfaces* **2019,** *11* (5), 4942-4953.
3. Cardona, C. M.; Li, W.; Kaifer, A. E.; Stockdale, D.; Bazan, G. C., Electrochemical considerations for determining absolute frontier orbital energy levels of conjugated polymers for solar cell applications. *Adv Mater* **2011,** *23* (20), 2367-71.
4. Shahi, M.; Atapattu, H. R.; Baustert, K. N.; Anthony, J. E.; Brill, J. W.; Johnson, S.; Graham, K. R., Probing transport energies and defect states in organic semiconductors using energy resolved electrochemical impedance spectroscopy. *Advanced Materials Interfaces* **2023**.
5. Durand, P.; Zeng, H.; Biskup, T.; Vijayakumar, V.; Untilova, V.; Kiefer, C.; Heinrich, B.; Herrmann, L.; Brinkmann, M.; Leclerc, N., Single Ether-Based Side Chains in Conjugated Polymers: Toward Power Factors of 2.9 mW m −1 K −2. *Advanced Energy Materials* **2021**.
6. Li, H.; DeCoster, M. E.; Ming, C.; Wang, M.; Chen, Y.; Hopkins, P. E.; Chen, L.; Katz, H. E., Enhanced Molecular Doping for High Conductivity in Polymers with Volume Freed for Dopants. *Macromolecules* **2019,** *52* (24), 9804-9812.
7. Peng, Z.; Ye, L.; Ade, H., Understanding, quantifying, and controlling the molecular ordering of semiconducting polymers: from novices to experts and amorphous to perfect crystals. *Mater Horiz* **2021**.
8. Jacobs, I. E.; Lin, Y.; Huang, Y.; Ren, X.; Simatos, D.; Chen, C.; Tjhe, D.; Statz, M.; Lai, L.; Finn, P. A.; Neal, W. G.; D'Avino, G.; Lemaur, V.; Fratini, S.; Beljonne, D.; Strzalka, J.; Nielsen, C. B.; Barlow, S.; Marder, S. R.; McCulloch, I.; Sirringhaus, H., High-Efficiency Ion-Exchange Doping of Conducting Polymers. *Adv Mater* **2021**, e2102988.
9. Gregory, S. A.; Menon, A. K.; Ye, S.; Seferos, D. S.; Reynolds, J. R.; Yee, S. K., Effect of Heteroatom and Doping on the Thermoelectric Properties of Poly(3-alkylchalcogenophenes). *Advanced Energy Materials* **2018,** *8* (34).
10. Pandolfi, R. J.; Allan, D. B.; Arenholz, E.; Barroso-Luque, L.; Campbell, S. I.; Caswell, T. A.; Blair, A.; De Carlo, F.; Fackler, S.; Fournier, A. P.; Freychet, G.; Fukuto, M.; Gursoy, D.; Jiang, Z.; Krishnan, H.; Kumar, D.; Kline, R. J.; Li, R.; Liman, C.; Marchesini, S.; Mehta, A.; N'Diaye, A. T.; Parkinson, D. Y.; Parks, H.; Pellouchoud, L. A.; Perciano, T.; Ren, F.; Sahoo, S.; Strzalka, J.; Sunday, D.; Tassone, C. J.; Ushizima, D.; Venkatakrishnan, S.; Yager, K. G.; Zwart, P.; Sethian, J. A.; Hexemer, A., Xi-cam: a versatile interface for data visualization and analysis. *J Synchrotron Radiat* **2018,** *25* (Pt 4), 1261-1270.
11. Lim, E.; Peterson, K. A.; Su, G. M.; Chabinyc, M. L., Thermoelectric Properties of Poly(3-hexylthiophene) (P3HT) Doped with 2,3,5,6-Tetrafluoro-7,7,8,8-tetracyanoquinodimethane (F4TCNQ) by Vapor-Phase Infiltration. *Chemistry of Materials* **2018,** *30* (3), 998-1010.
12. Ma, T.; Dong, B. X.; Grocke, G. L.; Strzalka, J.; Patel, S. N., Leveraging Sequential Doping of Semiconducting Polymers to Enable Functionally Graded Materials for Organic Thermoelectrics. *Macromolecules* **2020,** *53* (8), 2882-2892.
13. Patel, S. N.; Glaudell, A. M.; Peterson, K. A.; Thomas, E. M.; O'Hara, K. A.; Lim, E.; Chabinyc, M. L., Morphology controls the thermoelectric power factor of a doped semiconducting polymer. *Science Advances* **2017,** *3* (6), e1700434.
14. Jacobs, I. E.; D'Avino, G.; Lemaur, V.; Lin, Y.; Huang, Y.; Chen, C.; Harrelson, T. F.; Wood, W.; Spalek, L. J.; Mustafa, T.; O'Keefe, C. A.; Ren, X.; Simatos, D.; Tjhe, D.; Statz, M.; Strzalka, J. W.; Lee, J. K.; McCulloch, I.; Fratini, S.; Beljonne, D.; Sirringhaus, H., Structural and Dynamic Disorder, Not Ionic Trapping, Controls Charge Transport in Highly Doped Conducting Polymers. *J Am Chem Soc* **2022,** *144* (7), 3005-3019.

15. Thomas, E. M.; Popere, B. C.; Fang, H.; Chabinyc, M. L.; Segalman, R. A., Role of Disorder Induced by Doping on the Thermoelectric Properties of Semiconducting Polymers. *Chemistry of Materials* **2018,** *30* (9), 2965-2972.
16. Gregory, S. A.; Hanus, R.; Atassi, A.; Rinehart, J. M.; Wooding, J. P.; Menon, A. K.; Losego, M. D.; Snyder, G. J.; Yee, S. K., Quantifying charge carrier localization in chemically doped semiconducting polymers. *Nature Materials* **2021**.
17. Ponder, J. F., Jr.; Gregory, S. A.; Atassi, A.; Menon, A. K.; Lang, A. W.; Savagian, L. R.; Reynolds, J. R.; Yee, S. K., Significant Enhancement of the Electrical Conductivity of Conjugated Polymers by Post-Processing Side Chain Removal. *J Am Chem Soc* **2022**.
18. Shallcross, R. C.; Stubhan, T.; Ratcliff, E. L.; Kahn, A.; Brabec, C. J.; Armstrong, N. R., Quantifying the Extent of Contact Doping at the Interface between High Work Function Electrical Contacts and Poly(3-hexylthiophene) (P3HT). *The Journal of Physical Chemistry Letters* **2015,** *6* (8), 1303-1309.
19. Bubnova, O.; Berggren, M.; Crispin, X., Tuning the Thermoelectric Properties of Conducting Polymers in an Electrochemical Transistor. *Journal of the American Chemical Society* **2012,** *134* (40), 16456-16459.
20. Zevalkink, A.; Smiadak, D. M.; Blackburn, J. L.; Ferguson, A. J.; Chabinyc, M. L.; Delaire, O.; Wang, J.; Kovnir, K.; Martin, J.; Schelhas, L. T.; Sparks, T. D.; Kang, S. D.; Dylla, M. T.; Snyder, G. J.; Ortiz, B. R.; Toberer, E. S., A practical field guide to thermoelectrics: Fundamentals, synthesis, and characterization. *Applied Physics Reviews* **2018,** *5* (2), 021303.
21. Hisaaki Tanaka, K. K., Naoya Takekoshi, Hiroaki Mada, Hiroshi Ito, Yukihiro Shimoi, Hiromichi Ohta, Taishi Takenobu, Thermoelectric properties of a semicrystalline polymer doped beyond the insulator-to-metal transition by electrolyte gating. *Science Advances* **2020,** *6*.
22. Rivnay, J.; Mannsfeld, S. C.; Miller, C. E.; Salleo, A.; Toney, M. F., Quantitative determination of organic semiconductor microstructure from the molecular to device scale. *Chem Rev* **2012,** *112* (10), 5488-519.
23. Rivnay, J.; Noriega, R.; Kline, R. J.; Salleo, A.; Toney, M. F., Quantitative analysis of lattice disorder and crystallite size in organic semiconductor thin films. *Physical Review B* **2011,** *84* (4).
24. Rivnay, J.; Noriega, R.; Northrup, J. E.; Kline, R. J.; Toney, M. F.; Salleo, A., Structural origin of gap states in semicrystalline polymers and the implications for charge transport. *Physical Review B* **2011,** *83* (12).
25. Jiao, X.; Statz, M.; Lai, L.; Schott, S.; Jellett, C.; McCulloch, I.; Sirringhaus, H.; McNeill, C. R., Resolving Different Physical Origins toward Crystallite Imperfection in Semiconducting Polymers: Crystallite Size vs Paracrystallinity. *J Phys Chem B* **2020,** *124* (46), 10529-10538.
26. Cochran, J. E.; Junk, M. J. N.; Glaudell, A. M.; Miller, P. L.; Cowart, J. S.; Toney, M. F.; Hawker, C. J.; Chmelka, B. F.; Chabinyc, M. L., Molecular Interactions and Ordering in Electrically Doped Polymers: Blends of PBTTT and F4TCNQ. *Macromolecules* **2014,** *47* (19), 6836-6846.
27. Mildred Dresselhaus; Gene Dresselhaus; Stephen Cronin; Antonio Gomes; Filho, S., *Solid State Properties: From Bulk to Nano*. 2018.
28. Vezie, M. S.; Few, S.; Meager, I.; Pieridou, G.; Dorling, B.; Ashraf, R. S.; Goni, A. R.; Bronstein, H.; McCulloch, I.; Hayes, S. C.; Campoy-Quiles, M.; Nelson, J., Exploring the origin of high optical absorption in conjugated polymers. *Nat Mater* **2016,** *15* (7), 746-53.
29. Gasiorowski, J.; Hingerl, K.; Menon, R.; Plach, T.; Neugebauer, H.; Wiesauer, K.; Yumusak, C.; Sariciftci, N. S., Dielectric Function of Undoped and Doped Poly[2-methoxy-5-(3',7'-dimethyloctyloxy)-1,4-phenylene-vinylene] by Ellipsometry in a Wide Spectral Range. *J Phys Chem C Nanomater Interfaces* **2013,** *117* (42), 22010-22016.
30. Woollam, J. A. Ellipsometry Resources. https://www.jawoollam.com/resources.
31. Hwang, J.; Schwendeman, I.; Ihas, B. C.; Clark, R. J.; Cornick, M.; Nikolou, M.; Argun, A.; Reynolds, J. R.; Tanner, D. B., In situmeasurements of the optical absorption of dioxythiophene-based conjugated polymers. *Physical Review B* **2011,** *83* (19).

32. Hwang, J.; Tanner, D. B.; Schwendeman, I.; Reynolds, J. R., Optical properties of nondegenerate ground-state polymers: Three dioxythiophene-based conjugated polymers. *Physical Review B* **2003,** *67* (11).
33. Scholes, D. T.; Yee, P. Y.; McKeown, G. R.; Li, S.; Kang, H.; Lindemuth, J. R.; Xia, X.; King, S. C.; Seferos, D. S.; Tolbert, S. H.; Schwartz, B. J., Designing Conjugated Polymers for Molecular Doping: The Roles of Crystallinity, Swelling, and Conductivity in Sequentially-Doped Selenophene-Based Copolymers. *Chemistry of Materials* **2018,** *31* (1), 73-82.
34. Thomas, E. M.; Peterson, K. A.; Balzer, A. H.; Rawlings, D.; Stingelin, N.; Segalman, R. A.; Chabinyc, M. L., Effects of Counter-Ion Size on Delocalization of Carriers and Stability of Doped Semiconducting Polymers. *Adv. Electron. Mater.* **2020,** *6* (12), 2000595.
